# Upcycling hazardous waste into high-performance Ni/η-Al_2_O_3_ catalysts for CO_2_ methanation†

**DOI:** 10.1039/d4gc05217j

**Published:** 2025-02-07

**Authors:** Qaisar Maqbool, Hamilton Uchenna Aharanwa, Michael Stöger-Pollach, Günther Rupprechter

**Affiliations:** a Institute of Materials Chemistry, TU Wien Getreidemarkt 9/BC 1060 Vienna Austria guenther.rupprechter@tuwien.ac.at; b University Service Center for Transmission Electron Microscopy, TU Wien Stadionallee 2/057-02 1020 Vienna Austria

## Abstract

Transforming hazardous and difficult-to-process waste materials, like spent Ni-MH batteries and aluminium foil, into nanocatalysts (NCts) provides a sustainable solution for resource management and reducing environmental impact. This study demonstrates a novel approach by extracting nickel sulfate (NiSO_4_·*x*H_2_O) from battery waste and subsequently converting it into Ni(OH)_2_ hydrogel precursors using l-glutamic acid. Waste aluminium foil was processed into alumina (Al_2_O_3_), and combined with Ni(OH)_2_ to synthesize Ni/η-Al_2_O_3_ NCts with 4% and 8% Ni loading. Characterization through XRD/SAED, STEM/EFTEM, and EELS revealed a disordered cubic structure of η-Al_2_O_3_, with well-dispersed Ni particles, making it effective for CO_2_ hydrogenation. The 8-Ni/η-Al_2_O_3_ exhibited the best catalytic performance, with CH_4_ selectivity of 99.8% and space time yield (STY) of 80.3 mmol_CH_4__ g_cat_^−1^ h^−1^ at 400 °C. The CO_2_ methanation mechanism over Ni/η-Al_2_O_3_ NCts was further explored using *operando* DRIFTS aligned with GC + MS. The *operando* investigation suggested a preferential associative CO_2_ methanation pathway, involving sequential adsorption and hydrogenation of CO_2_ to hydrogen carbonates on Ni/η-Al_2_O_3_, and their transformation into formate and methoxy intermediates leading to methane. Finally, to complete the upcycling/recycling loop, the spent Ni/η-Al_2_O_3_ NCts were recycled into Ni and Al precursors. These findings underscore the potential of upcycling waste materials for synthesizing sustainable, high-performance NCts, and offer insights into the CO_2_ methanation mechanism.

## Abstract 2

Converting battery and aluminium waste to efficient Ni/η-Al_2_O_3_ methanation catalysts enables sustainable waste management and CO_2_ reduction.

## Graphical abstract

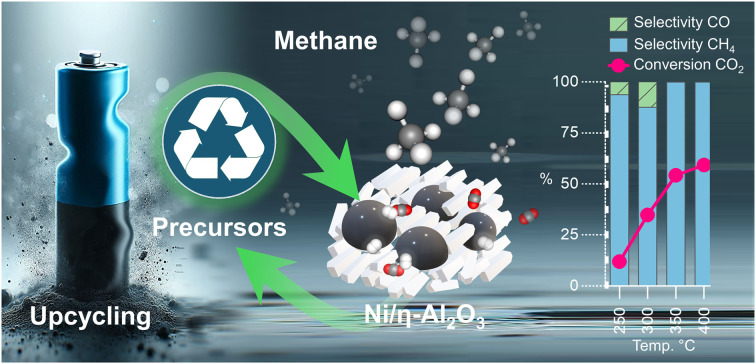

**Green foundation**

1. Hazardous waste (Ni-MH batteries and aluminum foil) is transformed to nanocatalysts (NCts) for CO_2_ hydrogenation. To close the loop, the spent catalyst was recycled into catalyst precursors, toward a circular economy and improving resource efficiency.

2. The waste-upcycled Ni/η-Al_2_O_3_ NCts achieve 99.8% CH_4_ selectivity and space time yield (STY) of 80.3 mmol_CH_4__ g_cat_^−1^ h^−1^ at 400 °C, enabling efficient CO_2_ conversion into sustainable synthetic fuel.

3. Future research may focus on optimizing catalyst recycling, reducing the catalyst operation temperature to enhance energy efficiency, using H_2_ from renewable sources, and exploring upscaling.

## Introduction

Power-to-Methane (PtM) technology, which uses the Sabatier reaction to convert CO_2_ and H_2_ into methane (CH_4_), was first commercialized in the early 2010s, providing means to store excess renewable energy in the existing natural gas infrastructure.^1–3^ Nowadays, this process utilizes H_2_ produced from water electrolysis, typically using solid oxide electrolysis cells (SOECs), and CO_2_ captured from flue gas, biomass, or other carbon-containing resources. PtM technology, when paired with carbon capture, presents a promising solution to mitigate climate change by reducing greenhouse gas emissions.^4^

Nevertheless, the economic feasibility of PtM plants remains a challenge, as the cost of synthesizing CH_4_ is currently several times higher than conventional natural gas.^5^ Some of the promising examples, including ‘MeGa-StoRE 2 – Optimising and Upscaling’ project in Denmark and the first PtM plant in Switzerland, which was built and operated at the ‘Institute for Energy Technology of the Hochschule für Technik Rapperswil (HSR-IET)’, both utilize Ni-based commercial catalysts for methanation.^6^ Still, most existing pilot plants have operated only for short periods, highlighting the need for improved efficiency to enhance economic competitiveness.^3^ Achieving this requires significant technical breakthroughs, particularly in the development of catalysts with high activity, selectivity, and durability, which are crucial for scaling up energy storage facilities.

Over the years, significant advancements have been made in the preparation of various materials with diverse structures and morphologies, leading to their successful application in catalysis, particularly for CO_2_ methanation.^7,8^ Among these, layered double hydroxides (LDHs) stand out as 2D materials with sheet-like structures that can be converted into mixed metal oxides. These oxides have proven to be highly effective catalysts for thermally driven CO_2_ methanation.^9,10^ Lima *et al.* prepared Ni–Al mixed metal oxides from LDHs, reporting the materials’ high CO_2_ conversion rate and CH_4_ selectivity, attributed to the high density of basic sites within the catalyst.^11^ In another study, 2D nickel@siloxene nanosheets presented a CO_2_ methanation rate of 100 mmol g_Ni_^−1^ h^−1^ with over 90% selectivity when nickel dwells specifically between the sheets of siloxane.^12^ Moreover, Gao *et al.* encapsulated Ni nanoparticles (NPs) within few-layer hexagonal boron nitride (h-BN) shells for syngas methanation. The confinement effect of the h-BN shells enhances the catalytic activity and stability of the Ni catalyst.^13^ Similarly, Zr,^14^ Cu/Zr/CdS,^15^ Ni/CeO_2_,^16^ and Co_3_O_4_/1D TiO_2_ nanowires^17^ based metal–organic frameworks (MOFs) with complex 2D/3D network structures have been explored for their catalytic efficiency, with notable success in enhancing surface area and electron transport, crucial for facilitating the CO_2_ to CH_4_ conversion process. Regardless of these advancements, research on 2D/3D materials for CO_2_ methanation remains limited.

Despite ongoing promising developments,^18–20^ a substantial gap remains in research concerning the sustainability of these catalytic processes. Most existing catalysts, particularly those based on Ni, Rh, Pd and Ru supported on materials such as Al_2_O_3_,^21–24^ TiO_2_,^22,25^ SiO_2_/SiC,^26,27^ ZrO_2_,^28^ and CeO_2_,^29,30^ rely heavily on synthetic reagents for their preparation. While these catalysts exhibit remarkable selectivity and stability for CH_4_ production, their reliance on non-renewable resources poses a challenge to the sustainability of the process. To address this issue, it is imperative to explore alternative, sustainable routes for catalyst preparation,^31^ ensuring that the entire process, from catalyst synthesis to CO_2_ methanation, aligns with the principles of environmental sustainability, in particular, UN Sustainable Development Goals (SDGs).^32^ This aspect, however, has been largely overlooked in current research, underscoring the need for a more comprehensive approach to sustainable catalyst production.

The increasing demand for Ni in PtM technologies for CO_2_ methanation highlights the potential benefits of extracting and recovering Ni from metal waste, such as spent nickel-metal hydride (Ni-MH) batteries.^33^ This approach not only addresses the challenge of upcycling difficult-to-process battery waste but also provides a valuable source of Ni metal salts, essential for Ni-based catalyst preparation in PtM applications. In Ni-MH batteries, nickel oxyhydroxide (NiOOH) is utilized in both electrodes. The reversible chemical reaction at the cathode mirrors that of nickel-cadmium (NiCd) cells (Ni(OH)_2_ + OH^−^ ↔ NiO(OH) + H_2_O + e^−^). However, the anode employs a hydrogen-absorbing alloy instead of cadmium (H_2_O + Me + e^−^ ↔ OH^−^ + MeH), where “Me” denotes the intermetallic compounds in the anode of an Ni-MH cell. Through processes such as acid leaching and selective precipitation, it is possible to recover high-purity Ni as NiSO_4_ from the cathode powder of spent Ni-MH batteries,^34,35^ making this method a promising avenue for sustainable resource recovery.

Still, Ni alone is insufficient for effective CO_2_ methanation, as it typically requires a compatible support material, such as metal oxides like Al_2_O_3_.^36^ In this context, aluminium (Al) can be extracted from waste aluminium foil, a common household item. In the current research, Ni was first recovered as NiSO_4_·*x*H_2_O from spent Ni-MH batteries and then transformed into Ni(OH)_2_ through l-glutamic acid and NaOH reduction. Concurrently, Al was extracted from waste aluminium foil and converted into Al_2_O_3_*via* NaOH treatment of AlCl_3_ to Al(OH)_3_. The resulting Ni(OH)_2_ was homogenized with Al_2_O_3_ to produce Ni/η-Al_2_O_3_ nanocatalysts (NCts). The physicochemical properties of NCts were thoroughly analyzed using advanced techniques such as high-resolution transmission electron microscopy (HR-TEM), energy-filtered transmission electron microscopy/scanning transmission electron microscopy coupled with electron energy loss spectrometry (EFTEM/STEM-EELS), selected area electron diffraction (SAED), temperature-programmed reduction (TPR), X-ray diffraction (XRD), and Brunauer–Emmett–Teller (BET) analysis. CO_2_ methanation was investigated at 1 bar in a continuous flow fixed-bed reactor, with kinetic measurements conducted at temperatures ranging from 250 °C to 550 °C. Additionally, mechanistic studies through *operando* DRIFTS aligned with GC + MS analyses provided insights into the surface activity of the Ni/η-Al_2_O_3_ NCts during CO_2_ methanation. Lastly, to close the upcycling loop by recycling, the spent Ni/η-Al_2_O_3_ NCts were successfully transformed back into Ni and Al precursors.

## Results and discussion

### Battery/Al-foil waste upcycling into nanocatalysts

**Fig. 1:**
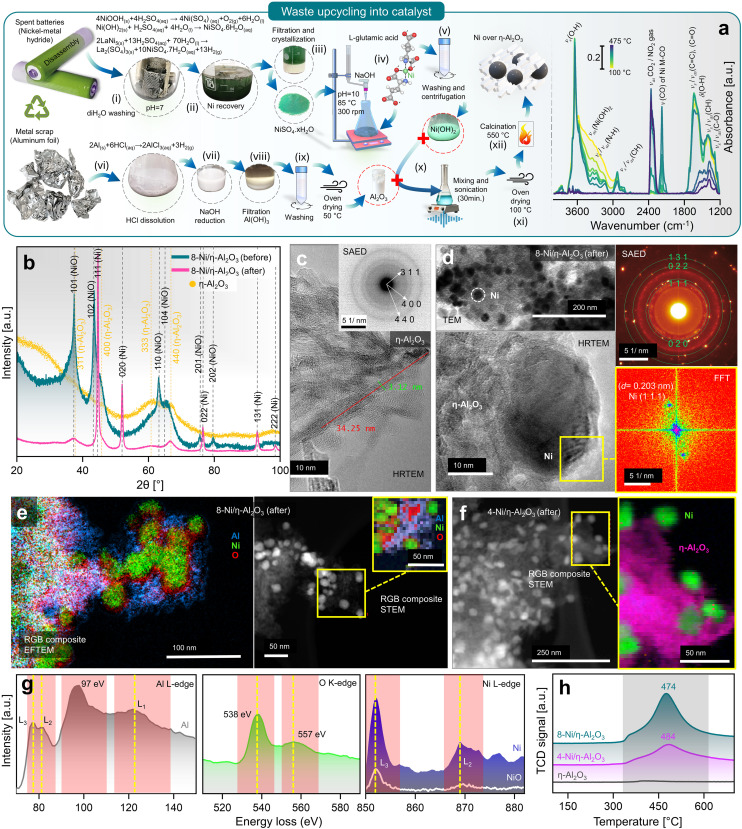
(a) Battery/aluminium waste upcycling and characterization of prepared nanocatalyst, with schematics elaborating the recovery of Ni as NiSO_4_·*x*H_2_O from spent Ni-MH batteries, Al_2_O_3_ from waste aluminium-foil, and recombination of Ni(OH)_2_ hydrogel complex and Al_2_O_3_ to obtain 4- (wt%) and 8-Ni/η-Al_2_O_3_ NCts (left), and *in situ* DRIFTS spectra during calcination showing thermal breakdown of Ni(OH)_2_ hydrogel complex at elevated temperature (100–475 °C) (right). (b) Crystal structure of η-Al_2_O_3_ and 8-Ni/η-Al_2_O_3_ NCts before and after CO_2_ methanation reaction (for 4-Ni/η-Al_2_O_3_ NCts, see Fig. S2†). (c) HRTEM of η-Al_2_O_3_ support. Inset: corresponding SAED pattern confirming the *hkl* (311), (400), and (440) of η-Al_2_O_3_. (d) TEM of 8-Ni/η-Al_2_O_3_ NCts after CO_2_ methanation showing homogenous Ni distribution over the η-Al_2_O_3_ support (top left), corresponding SAED pattern confirming the face-centred cubic crystal structure of Ni (top left), HRTEM of Ni lattice planes (bottom left) with FFT of the selected Ni *hkl* (111) (bottom right). (e) Color-coded elemental (Al, Ni, and O) mapping by EFTEM (left), and by STEM (right) of 8-Ni/η-Al_2_O_3_ NCts after CO_2_ methanation. (f) Color-coded elemental (pink = η-Al_2_O_3_, and green = Ni) mapping by STEM of 4-Ni/η-Al_2_O_3_ NCts after CO_2_ methanation. (g) EELS spectra corresponding to Al L_3,2_-edges, O K-edge, and Ni L_3,2_-edges. (h) H_2_-TPR spectra of 4, 8-Ni/η-Al_2_O_3_ NCts, and η-Al_2_O_3_ support.

Upcycling hazardous and difficult-to-process waste into valuable catalysts is a promising approach for sustainable resource management and reducing the environmental impact of waste disposal.^37^ This process also highlights the potential for recovering valuable metals, such as Ni and Al, from end-of-life products. In this context, the upcycling of spent battery and aluminium foil waste into nanocatalysts (NCts) is illustrated in Fig. 1a. Nickel sulfate hydrate (NiSO_4_·*x*H_2_O) was successfully extracted from spent Ni-MH batteries, primarily from the cathode powder, through a chemical process. The extraction and crystallization process from the cathode produced NiSO_4_ with high phase purity of ≈100%, as determined by XRF spectroscopy (Fig. S1†). This result aligns with the phase purity achieved in previously reported Ni-extraction methods.^34,35^ While NiSO_4_ can also be recovered from a mixture of cathode and anode materials, the resulting phase purity is lower (≈84%) due to contamination mainly from Co, Fe, La and Zn (Fig. S1†). The batteries were first disassembled, and the inner components were washed with diH_2_O to remove the KOH electrolyte, then dissolved in dilH_2_SO_4_. The nickel hydroxide Ni(OH)_2_ and nickel oxyhydroxide (NiOOH) (black powder) from cathode reacts with diH_2_SO_4_ to produce nickel sulfate-hydrate (NiSO_4_·*x*H_2_O), which was then filtered, heat-dried (saturated), and crystalized to obtain NiSO_4_·6H_2_O (used in this study). Additionally, the nickel-based alloy (LaNi_5_) from anode powder reacts with diH_2_SO_4_ to produce nickel sulfate-hydrate (NiSO_4_·*x*H_2_O), lanthanum sulfate (La_2_(SO_4_)_3_), and H_2_ gas. The La_2_(SO_4_)_3_ precipitates as a slag, while the blue-green coloured NiSO_4_·7H_2_O remains in solution, which was then filtered, heat-dried (saturated), and crystalized to obtain NiSO_4_·7H_2_O (not used in this study). The recovered La_2_(SO_4_)_3_ precipitates were also stored for future use.

Next, a 0.5 M solution of NiSO_4_·7H_2_O obtained from cathode upcycling was dissolved in diH_2_O, heated to 85 °C, and 0.25 M of l-glutamic acid was added. l-Glutamic acid has two carboxyl groups (–COOH) and one amino group (–NH_2_) with an isoelectric point of 3.2 (p*K*_a_ = 2.1, 4.3, and 9.7), which can act as a metal ion chelating agent to bind Ni ions.^38^ Furthermore, when 5 M NaOH is added, the pH of the mixture rises to 10, causing l-glutamic acid to exist as a doubly-negative anion, −OOC–CH(NH_2_)–(CH_2_)_2_–COO^−^.^39^ In this form, the carboxyl groups can donate electron pairs to Ni^2+^ ions, forming stable chelate complexes.^40^ This process results in the formation of Ni(OH)_2_, a hydrogel. The obtained l-glutamic acid-derived Ni(OH)_2_ hydrogel complex can easily attach to the substrate (*e.g.*, Al_2_O_3_ support) and can be used as a precursor for the preparation of various Ni-based NCts.

The *in situ* calcination of oven-dried (100 °C) Ni(OH)_2_ hydrogel complex was analyzed using DRIFT spectroscopy (Fig. 1a, right). The IR spectra revealed a dominant band at 3644 cm^−1^, with a broad shoulder between 3370–3550 cm^−1^, corresponding primarily to non-hydrogen bound and hydrogen bound OH groups of Ni(OH)_2_.^41^ Bands associated with N–H and C–H stretching vibrations of the doubly-negative anion were observed in the range of 2820–3346 cm^−1^.^42^ A sharp band at 2177 cm^−1^ was attributed to Ni bound to carbon and oxygen (metal carbonyls, M-CO).^43–45^ The region between 1200–1800 cm^−1^ was mainly indicative of C–O, CH, O–H, C=C, and C=O groups within the Ni(OH)_2_ hydrogel complex.^42^ During the temperature increase, decomposition between 100–300 °C proceeded slowly. However, temperatures above 300 °C significantly accelerated the release of CO_2_ (2360 cm^−1^) and H_2_O, primarily due to the breakdown of hydroxyl and carbon species. Although nitrogen from the Ni(OH)_2_ hydrogel complex might have been released as NO_2_, it was not detected in the CO_2_ background. The *in situ* calcination confirms that the transformation of l-glutamic acid-derived Ni(OH)_2_ hydrogel complex to NiO_2_ mainly occurs at *T* > 300 °C.

Furthermore, Al_2_O_3_ was obtained from waste aluminium foil, which is typically recycled to produce materials of the same class. The utilization of low-cost metal waste to produce high-value, functionalized materials (*e.g.*, NCts) has not yet been fully recognized. Through a two-step process, the aluminium foil was first dissolved in HCl, and the AlCl_3_ solution was then reduced with 5 M NaOH to obtain Al(OH)_3_ pellets. The Al(OH)_3_ were dried at 50 °C to obtain Al_2_O_3_. NCts containing 4 and 8 (wt%) Ni were synthesized by combining Ni(OH)_2_ and Al_2_O_3_. The two components were sonicated, then oven-dried to remove excess water. The dried pellets were calcined at 550 °C to obtain the NCts (for more details see the Methods).

### Characterization of NCts

The XRD analysis of the as-synthesized NCts, before and after CO_2_ hydrogenation reaction (Fig. 1b and Fig. S2a†) provides valuable insights into their structural properties. The as-synthesized Al_2_O_3_ (Fig. 1b) revealed broad diffraction peaks primarily corresponding to *hkl* of (311), (400), (333), and (440). These lattice planes are characteristic of the disordered and cubic spinel crystal structure of η-Al_2_O_3_ with the space group *Fd*3̄*m*.^46^ The broadness of these reflections suggests a small crystallite size of η-Al_2_O_3_. When NiO was introduced into the η-Al_2_O_3_ framework to form the 4 (wt%) and 8-Ni/η-Al_2_O_3_ NCts, the XRD patterns (Fig. 1b and Fig. S2†) showed broad *hkl* at (101), (102), (110), (104), (201), and (202), which are indicative of the hexagonal crystal structure of NiO with the space group *R*3̄*m*.^47,48^ However, after CO_2_ hydrogenation reaction, the NiO underwent a structural transformation, as evidenced by the appearance of sharp reflections at (111), (020), (022), (131), and (222). These Bragg peaks correspond to the face-centred cubic crystal structure of Ni with the space group *Fm*3̄*m*.^49,50^ Notably, the crystal structure of η-Al_2_O_3_ remained unaffected both before and after thermal (≈550 °C) CO_2_ hydrogenation reaction, suggesting η-Al_2_O_3_ resistance to phase transformation.^51^ Moreover, by analysing the two most intense Ni peaks at 2*θ* = 44.65° and 52.1° (Fig. S2b†) and applying the Debye–Scherrer equation,^52^ we calculated the average crystallite (grain) sizes. The results show an average crystallite size of 27.4 nm for 4-Ni/η-Al_2_O_3_ and 28.9 nm for 8-Ni/η-Al_2_O_3_. These values indicate that there is no significant difference in Ni crystallite size between the two NCts.

The morphology and crystal structure were further confirmed by HRTEM and selected area electron diffraction (SAED).^53^ The η-Al_2_O_3_ appears (Fig. 1c and Fig. S3†) as needle-like structures of *l* = ≈34 nm and ∅ = ≈3 nm. The SAED (inset of Fig. 1c) showed three distinct diffraction rings. The measured *d*-spacings (0.251, 0.196, and 0.140 nm) and the rotational average plot of the SAED (Fig. S4†) confirm the (311), (400), and (440) planes of the disordered η-Al_2_O_3_ cubic spinel crystal structure. The cubic spinel structure, commonly found in phases of Al_2_O_3_, belongs to the space group *Fd*3*m*. In a typical spinel composition, denoted as AB_2_O_4_, ‘A’ and ‘B’ represent different atomic species, such as Mg and Al in the spinel mineral (MgAl_2_O_4_).^54^ In a normal spinel structure, oxygen (O) atoms form a face-centred cubic (f.c.c.) sublattice, while the ‘A’ and ‘B’ atoms occupy specific interstices within this sublattice. Specifically, the O atoms occupy the (e) sites of the *Fd*3*m* space group, with ‘A’ and ‘B’ atoms residing in the tetrahedrally coordinated (a) sites and the octahedrally coordinated (d) sites, respectively.^55,56^ In one unit cell of spinel, there are 8 formula units of AB_2_O_4_, containing 8 A atoms, 16 B atoms, and 32 O atoms. In the case of cubic Al_2_O_3_, which has the spinel structure, there are 21 Al atoms per unit cell to fill the 8(a) and 16(d) sites. This leaves 2 vacancies, which can be distributed across the 8(a) and 16(d) interstices in various ways, introducing a degree of disorder into the structure. In γ-Al_2_O_3_, all 16 octahedrally coordinated (d) interstices of the O sublattice are fully occupied, similar to a normal spinel structure, with the remaining 5 Al ions distributed among the 8 tetrahedrally coordinated (a) interstices.^57^ In disordered η-Al_2_O_3_, Al atoms not only occupy the (a), (d), (f), and (c) sites typical of the normal spinel structure but also a small fraction of the 48 tetrahedrally coordinated (g) sites and octahedrally coordinated (c) sites within the *Fd*3*m* space group.^58^ However, the precise arrangement of Al atoms within the disordered η-Al_2_O_3_ unit cell is still an ongoing discussion.^59^

These metastable Al_2_O_3_ phases share several common characteristics, such as (1) they are cation-deficient spinel analogues, characterized by the distribution of Al atoms in octahedral and tetrahedral sites, with phase transitions between these forms being pseudomorphic despite marked lattice distortions in θ and κ phases,^60^ (2) the cubic cell, or its equivalent, has a consistent volume of approximately 7.93 Å^3^ across different varieties, (3) disorder occurs at various scales: in the γ, η, and θ forms, the Al sites are partially occupied without observable ordering; in the *δ* form, the crystal cell is fully ordered but owns a complex intergrowth from two main crystallographic variants,^61,62^ and (4) the crystallite size does not exceed a few tens of nanometers, which is a critical feature for applications of metastable aluminas, such as catalytic supports and absorbents, due to their high specific surface area.^63^ Therefore, we expect η-Al_2_O_3_ to be a perfect support for Ni in CO_2_ methanation, which will be discussed below.

Fig. 1d (top left) and Fig. S5, S6† demonstrate the uniform distribution of pristine NiO and Ni within the η-Al_2_O_3_ matrix, both before and after thermal CO_2_ hydrogenation. The Ni particles (4 and 8-Ni/η-Al_2_O_3_), as shown in Fig. S6,† were observed to be 18 to 39 nm in size and were evenly dispersed throughout the η-Al_2_O_3_ matrix, with no evidence of agglomeration. The SAED pattern (Fig. 1d, top right) revealed diffraction spots corresponding to the cubic crystal structure of Ni. Additionally, the Fast Fourier Transform (FFT) analysis (Fig. 1d, bottom right) of the HRTEM image (Fig. 1d, bottom left) confirmed the lattice spacing (*d* = 0.203 nm) of Ni (111). Post-thermal CO_2_ hydrogenation, the 4 and 8-Ni/η-Al_2_O_3_ NCts were further analyzed using EFTEM, STEM, and EELS. Elemental mapping through EFTEM (Fig. 1e, left), combined with EELS, confirmed the distribution of metallic Ni, the presence of O traces at the Ni/η-Al_2_O_3_ interfaces, and O enrichment within the η-Al_2_O_3_ lattice. Notably, the RGB composite STEM image (Fig. 1e, right) also highlighted O traces at the Ni/η-Al_2_O_3_ interfaces, suggesting potential anchoring of Ni onto the η-Al_2_O_3_ lattice. Similarly, the RGB composite STEM image of the 4-Ni/η-Al_2_O_3_ NCts (Fig. 1f) post-thermal CO_2_ hydrogenation also indicated the distribution of metallic Ni across the η-Al_2_O_3_ surface.

The disordered cubic crystal structure of η-Al_2_O_3_ has a lower symmetry than other Al phases (*e.g.* α-Al_2_O_3_)^64^ which can lead to different electronic band structures and, therefore, electron energy loss spectroscopy (EELS) analysis was necessary to perform, as shown in Fig. 1g. EELS of Al reflect the complex electronic structure of η-Al_2_O_3_, influenced by the mixed coordination of Al atoms and the degree of disorder in the material. The peaks at 77 and 81 eV, with 4.7 eV and 5.8 eV full width at half maximum (FWHM), respectively, correspond to the transitions from the 2p_3/2_ (Al–L_3_) and 2p_1/2_ (Al–L_2_) core levels to the unoccupied states above the Fermi level.^65^ The less intense and broad nature of these peaks indicates that the transitions are not sharply defined, indicating a degree of disorder in the local environment of the Al atoms which can be attributed to the mixed coordination states (tetrahedral and octahedral) of Al in η-Al_2_O_3_ lattice.^66^ The most intense peak at 97 eV is indicative of a strong transition to a specific unoccupied state while the broad and least intense peak at 122 eV corresponds to the Al–L_1_ edge. This feature represents higher energy transitions, possibly into more delocalized states further up in the conduction band. The broadness of this peak indicates that the final states available for these transitions are more spread out in energy (eV), reflecting the complex and somewhat disordered structure of η-Al_2_O_3_. This broad peak might also be influenced by multiple scattering events or by transitions involving more complex electronic states that are less localized.^65^ Additionally, the ratio of the most intense peak at 97 eV to the other peaks is quite different from that observed in other polymorphs of Al_2_O_3_,^67–71^ highlighting the unique electronic environment and structural characteristics of η-Al_2_O_3_.

O–K edge of η-Al_2_O_3_ (Fig. 1g) shows a more intense peak at 538 eV, corresponding to the transition of electrons from the O 1s core level to the unoccupied states in the conduction band, primarily the O 2p states that are hybridized with the Al 3s and 3p states.^72–74^ The relatively sharp and intense nature of this peak indicates a well-defined electronic transition, suggesting a strong hybridization between the O and Al atoms. The second peak, which is broader and less intense, centred at ≈557 eV, corresponds to transitions into more delocalized or higher energy states in the conduction band which possibly involve a combination of hybridized orbitals, including higher energy Al orbitals and possibly more delocalized states within the O network.^75,76^ The lower intensity compared to the first peak implies that there are fewer available states with O 2p character at this higher energy level.

The Ni–L edge in EELS (Fig. 1g) for metallic Ni exhibits three distinct peaks: a sharp and highly intense peak at 852 eV corresponding to the Ni–L_3_ edge, a broader and less intense peak at 869 eV corresponding to the Ni–L_2_ edge, and a third broad and least intense peak at 876 eV.^77^ These features arise from electronic transitions of electrons from the Ni 2p core levels (2p_3/2_ and 2p_1/2_) to unoccupied d-states. The Ni–L_3_ edge is more intense and sharper due to the higher density of available unoccupied d-states and stronger transition probabilities associated with the 2p_3/2_ to d transitions.^78,79^ The Ni–L_2_ edge, being broader and less intense, results from 2p_1/2_ to d-transitions,^80^ which have different selection rules and lower transition probabilities. The third peak at 876 eV may be attributed to higher energy transitions or multiple scattering effects that broaden and diminish its intensity. In contrast, NiO displays a less distinct separation between the first two peaks and overall lower intensities across all three peaks. This difference is due to the distinct electronic structure of NiO, where Ni exists in a +2-oxidation state, leading to filled lower Hubbard bands and empty upper Hubbard bands influenced by strong electron correlations and charge transfer from O.^81,82^ The increased hybridization between Ni and O in NiO results in broader and less pronounced peaks, as the transitions involve more complex interactions and a redistribution of spectral weight. Additionally, the presence of charge-transfer satellites in NiO can further obscure the distinction between the Ni–L_3_ and Ni–L_2_ edges,^83,84^ reducing the peak intensity and clarity compared to metallic Ni. Therefore, the EELS features reflect the fundamental differences in the electronic environments and bonding characteristics of metallic nickel and NiO. However, EELS data for 4-Ni/η-Al_2_O_3_ and 8-Ni/η-Al_2_O_3_ after CO_2_ methanation, including Ni L-edge, differ only in intensity due to Ni loading (Fig. S7†), confirming that no significant structural or electronic differences beyond Ni content.

The Temperature Programmed Reduction (TPR) profiles of 8 wt% and 4 wt% Ni over η-Al_2_O_3_ reveal distinct peaks (Fig. 1h), with the 8% Ni sample exhibiting a broad and intense peak at 474 °C, while the 4% Ni sample shows a similar, though less intense, peak slightly shifted to 484 °C. The TPR peaks represents the thermal energy required to reduce Ni^2+^ in the NiO lattice to Ni^0^, with the hexagonal NiO phase (space group *R*3̄*m*) transitioning to the more stable cubic Ni phase (space group *Fm*3̄*m*) upon reduction.^85,86^ The differences in peak intensity and position reflect variations in the NiO particle size, distribution, and interaction with the η-Al_2_O_3_ support, all of which influence the reduction kinetics and the overall reduction process.^87^ The slight temperature shift can be attributed to the concentration of NiO and its interaction with the η-Al_2_O_3_ support. Higher NiO loading (8%) results in a more extensive and interconnected NiO phase, leading to a more pronounced reduction peak due to the larger quantity of NiO being reduced. The slightly lower reduction temperature (474 °C) for 8% NiO suggests that the NiO having higher coverage of the support surface may facilitate easier reduction, likely due to the stronger interactions between NiO particles, which promote faster electron transfer and reduce the energy barrier for the reduction process.^88^ The shift to a slightly higher reduction temperature (484 °C) in the 4% Ni sample suggests that the more dispersed NiO particles on the η-Al_2_O_3_ surface require slightly more energy to reduce. This may be due to the higher surface energy and the stronger metal-support interaction (MSI),^89^ making NiO particles more resistant to reduction.

The BET analysis, as shown in Fig. S8,† provides the specific surface area of the NCts, which is crucial for understanding their catalytic properties and overall performance. The pure η-Al_2_O_3_ support exhibits a surface area (SBET) of 53.41 m^2^ g^−1^, which increases significantly upon NiO loading, reaching 159.02 m^2^ g^−1^ for 4-Ni/η-Al_2_O_3_ and 226.82 m^2^ g^−1^ for 8-Ni/η-Al_2_O_3_, indicating that higher NiO loadings increase SBET. BJH analysis reveals that pore sizes decrease from 9.68 nm in η-Al_2_O_3_ to 6.85 nm and 6.42 nm for 4-Ni/η-Al_2_O_3_ and 8-Ni/η-Al_2_O_3_, respectively, suggesting partial pore blocking by NiO. However, the overall increase in SBET highlights that the introduction of NiO compensates for this reduction. The significant increase in SBET upon Ni loading (from 159.02 m^2^ g^−1^ for 4 wt% Ni to 226.82 m^2^ g^−1^ for 8 wt% Ni) cannot be explained solely by the NiO particles. As calculated (ESI Note 1†), the specific surface area contribution of NiO particles is not significant, even for the smallest particle sizes observed (2.5 nm, Fig. S5†). Instead, the enhancement in SBET is likely due to modifications of the η-Al_2_O_3_ support induced by the Ni loading process. These modifications may involve structural rearrangements, increased surface roughness, or interfacial effects, which enhance the overall surface area of the NCts. Further detailed investigations are required to confirm this. The type H3 isotherm hysteresis recorded during the BET analysis (Fig. S8†) further elucidates the physisorption behaviour of both the Ni/η-Al_2_O_3_ NCts and the η-Al_2_O_3_ support. The hysteresis loop, which comprises of an adsorption isotherm (type II) and a desorption isotherm,^90^ indicates the interaction of N_2_ with the NCts surfaces. Initially, the adsorption isotherm shows N_2_ forming a monolayer on the sample, leading to a strong increase in adsorption due to direct interaction with the porous surface. As the relative pressure (*p*/*p*^0^) rises, the isotherm bends, indicating a slower rate of adsorption attributed to the formation of a multilayer, which interacts less strongly with the NCts surface.^91^ At *p*/*p*^0^ ≈ 1, the multilayer reaches its critical film thickness, causing N_2_ molecules to interact and fill the NCts pores, a phenomenon known as capillary condensation.^92^ Upon reducing the relative pressure, N_2_ desorbs from the NCts pores through evaporation, a process distinct from capillary condensation which involves the condensation of vapor in small pores due to capillary forces, as evidenced by the hysteresis loop in the isotherm (Fig. S8†), further suggesting the mesoporous nature of the NCts.

### CO_2_ methanation performance of nanocatalysts

**Fig. 2:**
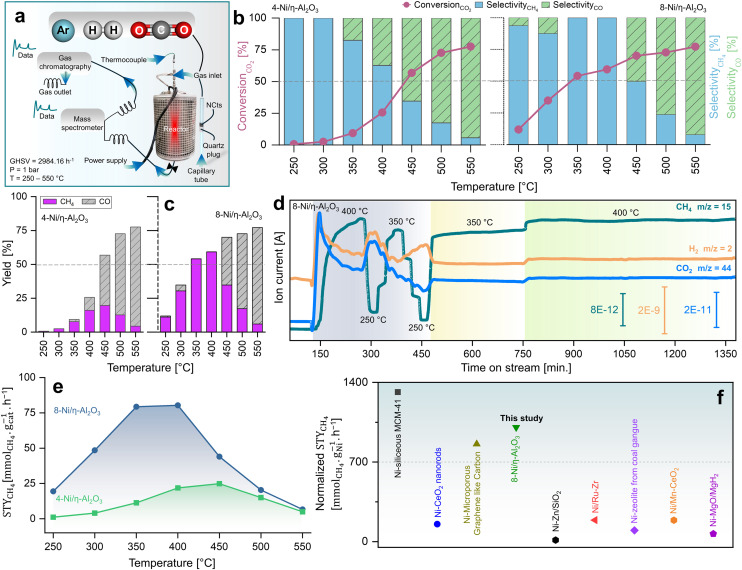
Kinetic measurements of CO_2_ methanation. (a) Reactor design for CO_2_ hydrogenation reaction at *p* = 1 bar and *T* = 250–550 °C operated at GHSV of 2984.16 h^−1^ and a CO_2_ : H_2_ ratio of 1 : 4. (b) Catalytic performance of 4 and 8-Ni/η-Al_2_O_3_ NCts in terms of CO_2_ conversion (%), CO and CH_4_ selectivity (%) and (c) CO/CH_4_ yield (%). (d) MS spectra showing stable CH_4_ signal (fragment as CH_3_ ion, *m*/*z* = 15), for both abrupt and prolonged (400 and 600 min.) thermal (350–400 °C) treatments. (e) STY mmol_CH_4__ g_cat_^−1^ h^−1^ at 250–350 °C of 4 and 8-Ni/η-Al_2_O_3_ NCts. (f) comparison of STY (mmol_CH_4__ g_Ni_^−1^ h^−1^) of best performing NCts (8-Ni/η-Al_2_O_3_) with Ni based NCts from literature.^97–104^ The literature STY_CH_4__ values were normalized to the same Ni content (%), pressure (1.01325 bar), and temperature (400 °C) for consistency. See Table S1† for more details.

Fig. 2a illustrates the setup used to measure the catalytic performance of the Ni/η-Al_2_O_3_ NCts at a gas hourly space velocity (GHSV) of 2984.16 h^−1^ and a CO_2_ : H_2_ ratio of 1 : 4. All NCts displayed a consistent increase in CO_2_ conversion with rising temperatures from 250 °C to 550 °C, achieving an overall CO_2_ conversion of 77% for both 4 and 8-Ni/η-Al_2_O_3_ (Fig. 2b). Notably, the NCts with higher Ni loading (8-Ni/η-Al_2_O_3_) significantly enhanced CH_4_ selectivity, reaching a maximum of 99.8% (Fig. 2b) and achieving CH_4_ yields of 54% and 59% at 350 °C and 400 °C, respectively (Fig. 2c). However, CH_4_ selectivity dropped to 49% at 450 °C and further declined to 8% at 550 °C (Fig. 2b), likely due to the thermodynamic favourability of the Reverse Water Gas Shift (RWGS) reaction (CO_2_ + H_2_ → CO + H_2_O, Δ*H* = +9 kcal mol^−1^),^93^ which produced nearly 70% CO as the side product at these higher temperatures (Fig. 2c). Interestingly, at a lower temperature of 300 °C, the CH_4_ yield of 4-Ni/η-Al_2_O_3_ was 2.5%, whereas it was significantly higher at 30% for 8-Ni/η-Al_2_O_3_ (Fig. 2c), underscoring the critical role of available active Ni sites over the η-Al_2_O_3_ in promoting CO_2_ hydrogenation toward the desired methanation pathway. Catalysts under CO_2_ methanation can deactivate over time or upon strong temperature changes,^94^ possibly due to coke formation,^95^ or sintering.^96^ Therefore, the stability of the best-performing NCts, 8-Ni/η-Al_2_O_3_, was rigorously tested, as shown in Fig. 2d. The NCts was subjected to significant temperature fluctuations (250 °C → 400 °C → 250 °C → 350 °C → 250 °C → 350 °C), held at 350 °C for 300 minutes, and then maintained at 400 °C for 600 minutes. Despite these challenging conditions, 8-Ni/η-Al_2_O_3_ demonstrated remarkable stability (Fig. 2d), with no signs of deactivation or decline in CH_4_ productivity, confirming its resilience under both abrupt and prolonged thermal treatments. Additionally, calculating the Space–Time Yield (STY) in CO_2_ methanation is crucial for assessing the efficiency and productivity of the NCts, which is vital for process optimization and potential scale-up. As expected, the 8-Ni/η-Al_2_O_3_ exhibited the highest STY, recording 79.3 mmol_CH_4__ g_cat_^−1^ h^−1^ at 350 °C and 80.3 mmol_CH_4__ g_cat_^−1^ h^−1^ at 400 °C, as shown in Fig. 2e. In contrast, the STY for 4-Ni/η-Al_2_O_3_ was significantly lower, with values of 11.3 mmol_CH_4__ g_cat_^−1^ h^−1^ at 350 °C and 21.8 mmol_CH_4__ g_cat_^−1^ h^−1^ at 400 °C. Even at the lower temperature of 250 °C, 8-Ni/η-Al_2_O_3_ achieved a STY of 19.4 mmol_CH_4__ g_cat_^−1^ h^−1^. The CO_2_ methanation performance of 8-Ni/η-Al_2_O_3_ was also compared with literature data^97–104^ (Fig. 2f and Table S1†), where it was found to be highly competitive. The promising CO_2_ methanation performance of Ni/η-Al_2_O_3_ can be attributed to several key factors. The disordered η-Al_2_O_3_ support plays a crucial role by enhancing the activity of Ni for CO_2_ methanation. This disordered structure likely facilitates better adsorption of CO_2_, creating more favorable sites where CO_2_ molecules/intermediates can interact stronger with the active Ni sites. This can also facilitate electron transfer processes, which are crucial for the activation of CO_2_ molecules during the methanation reaction, stabilizing the Ni particles, preventing them from agglomerating and thus maintaining a high dispersion of active sites (Fig. 1d–f). Additionally, the well-dispersed and homogenous size of Ni particles, coupled with their high surface area (Fig. S5†), ensures a sufficient number of active sites are available for the H_2_ binding. Another important factor is the low reducibility of Ni (<500 °C) over the η-Al_2_O_3_ support, which aids in the activation of Ni at relatively lower temperatures. To support these rationalized reasons for the superior CO_2_ hydrogenation performance of Ni/η-Al_2_O_3_, we have conducted *operando* DRIFTS studies,^105^ as detailed below.

### *Operando* insights into CO_2_ methanation through DRIFTS/GC + MS

**Fig. 3:**
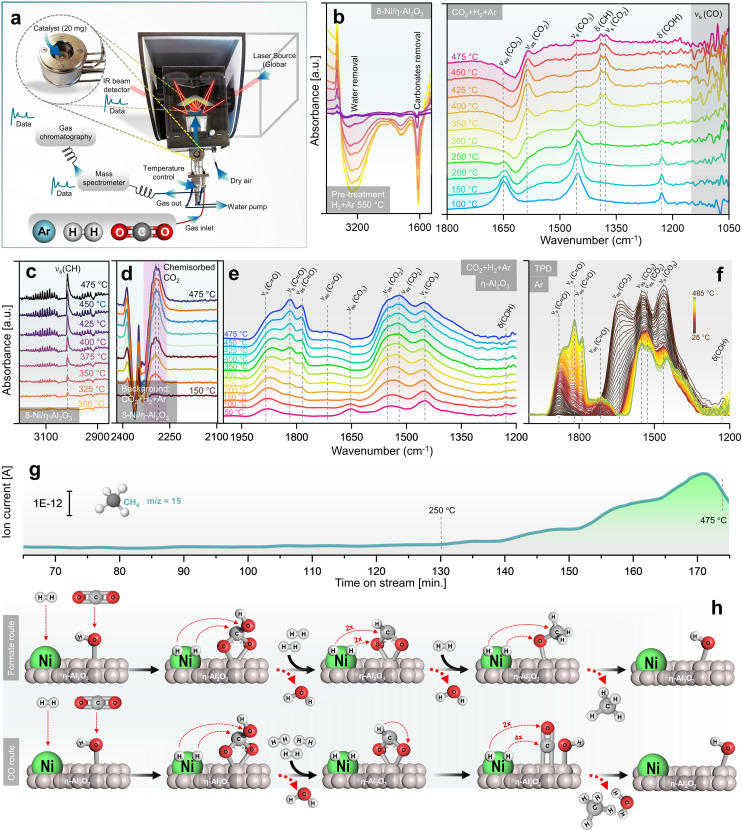
Rationalized mechanistic study of CO_2_ methanation by operando DRIFTS/GC + MS. (a) Experimental setup showing DRIFTS along catalytic performance measurements *via* GC + MS, under operando conditions. (b) DRIFTS spectra collected during the pre-treatment of NCts in a 5% H_2_ in Ar atmosphere at 550 °C, showing IR peaks originating from removal of carbonaceous species and surface adsorbed H_2_O (left), and DRIFTS spectra of 8-Ni/η-Al_2_O_3_ NCts exposed to a gas mixture of 5% CO_2_, 20% H_2_, and 75% Ar in a temperature range of 100–475 °C, showing formation of mainly hydrogen carbonates, formate and methoxy species (right). (c) DRIFTS spectra of CH_4_ formation in gas phase over the active 8-Ni/η-Al_2_O_3_ NCts. (d) DRIFTS spectra of chemisorbed CO_2_ over 8-Ni/η-Al_2_O_3_ NCts, observed taking CO_2_/H_2_/Ar as background during heat treatment (150–475 °C). (e) DRIFTS spectra of η-Al_2_O_3_ support exposed to a gas mixture of 5% CO_2_, 20% H_2_, and 75% Ar in a temperature range of 50–475 °C, showing formation of mainly hydrogen carbonates species. (f) Temperature programmed desorption (TPD) measured between 25–485 °C of (e). (g) MS spectra acquired simultaneously to the DRIFTS measurements, showing a linear increase in CH_4_ (fragment as CH_3_ ion, *m*/*z* = 15) formation with temperature. (h) CO_2_ methanation pathways over Ni/η-Al_2_O_3_ NCts, hypothesized based on observations in (b)–(g).

A mechanistic study of CO_2_ methanation over Ni/η-Al_2_O_3_ NCts was conducted using DRIFTS alongside catalytic performance measurements *via* GC + MS, in an *operando* setup^106^ (Fig. 3a). The calcined NCts underwent a pre-treatment process in a 5% H_2_ in Ar atmosphere at 550 °C, which effectively removed surface contaminants such as carbonates and H_2_O (Fig. 3b), thereby activating the NCts. This pre-treatment was crucial for ensuring the NCts’ surface was clean and ready for the subsequent CO_2_ methanation reaction.

Following the H_2_ pretreatment, the 8-Ni/η-Al_2_O_3_ NCts were exposed to a gas mixture of 5% CO_2_, 20% H_2_, and 75% Ar across a temperature range of 100–475 °C. Strong infrared bands were observed at 1648 cm^−1^ (*ν*_as_(CO_3_)), 1452 cm^−1^ (*ν*_s_(CO_3_)), and 1228 cm^−1^ (*δ*(COH)), which can be attributed to hydrogen carbonate species^107–109^ adsorbed on η-Al_2_O_3_ (Fig. 3b). As the temperature increased to 250 °C, peaks at 1585 cm^−1^ (*ν*_as_(CO_2_)), 1390 cm^−1^ (*δ*(CH)), and 1377 cm^−1^ (*ν*_s_(CO_2_)) began to grow, suggesting the formation of formate species,^110^ which are often considered as intermediates in CH_4_ formation. At higher temperatures, the formation of methoxy species (*ν*_s_(CO)) was evident at 1050–1100 cm^−1^. The IR signals in this region were sharp and transient, indicating rapid formation and transformation of methoxy species to CH_4_.^111^ This suggests that methoxy species are quickly formed and rapidly converted to CH_4_, highlighting the dynamic nature of the reaction at elevated temperatures. Consistent with previous studies, bicarbonate species are progressively reduced by H^+^ spillover, transforming into formate and methoxy species, which ultimately form CH_4_.^112^ At temperatures exceeding 300 °C, the formation of gas-phase CH_4_ was confirmed by the growth of the *ν*_s_(CH) peak at 3015 cm^−1^ (Fig. 3c). Additionally, bands at 2293 cm^−1^ and 2284 cm^−1^ were observed, which are related to chemisorbed CO_2_ on the Lewis acid sites^113^ of η-Al_2_O_3_ (Fig. 2d). The presence of chemisorbed CO_2_ on these Lewis acid sites may play an essential role in facilitating CO_2_ dissociation and subsequent hydrogenation.^114^ The formation of hydrogen carbonate species between 1400–1600 cm^−1^ was also noted (Fig. 3e). Interconversion of carbonate to carbonyl species (*v*CO_2_ ⇌ *v*C=O) specific to the disordered η-Al_2_O_3_ phase was observed.^115^ These species permanently accumulated on the η-Al_2_O_3_ surface as the temperature increased, persisting even after the CO_2_/H_2_ cut-off and temperature-programmed desorption (TPD) under Ar up to 485 °C (Fig. 3f). Clearly, their transformation required active sites from Ni for further hydrogenation and C–O/C=O bond breaking.^110^ The CH_4_ formation MS signal, parallel to the DRIFTS measurements, showed a linear increase in CH_4_ formation with temperature, particularly from 350–475 °C (Fig. 3g). While the study provides support for the evolution of intermediates and products, further confirmation of the role of observed surface species as active intermediates (rather than spectators) would require isotopic labelling studies *via* steady-state isotopic transient kinetic analysis (SSITKA)-DRIFTS-MS^116,117^ or, alternatively, modulation excitation spectroscopy (MES).^105,118–123^

Finally, the *operando* DRIFTS analysis (Fig. 3a–g) revealed that the CO_2_ hydrogenation pathway likely follows an associative CO_2_ methanation mechanism, involving the sequential adsorption and hydrogenation of CO_2_ on the NCts surface to produce CH_4_. The proposed formate route (Fig. 3h) begins with Ni and η-alumina adsorbing H_2_ and CO_2_ molecules, respectively. The CO_2_ interacts with OH ions previously adsorbed by η-Al_2_O_3_, forming a bidentate hydrogen carbonate species, which is further hydrogenated to form a bidentate formate. The amphoteric nature of η-Al_2_O_3_ plays a critical role in this process. Its surface Lewis acid sites facilitate the activation of CO_2_ molecules,^124^ while its basic hydroxyl groups enhance adsorption and stabilize intermediate species, such as hydrogen carbonates and formates.^125^ This dual functionality may enable η-Al_2_O_3_ to serve as an effective support, promoting CO_2_ activation and ensuring efficient progression along the methanation pathway. This formate is hypothesized to selectively hydrogenate into a methoxy species before CH_4_ is released from the NCts.^126^ At lower Ni loading (4-Ni/η-Al_2_O_3_), the active sites appear to favour CO formation through initial CO_2_ activation, leading to CH_3_O species or carbonyl formation and higher CO yields. However, with increased Ni loading (8-Ni/η-Al_2_O_3_), the availability of active sites may facilitate further hydrogenation of CO, promoting the conversion of methoxy species into CH_4_. The alternative CO route (Fig. 3d) follows similar steps for bidentate formate formation but involves formate decomposition to CO,^127^ which can either be desorbed or further hydrogenated to produce CH_4_ and H_2_O. The transition from bicarbonate to formate species is suggested to involve C–O bond cleavage *via* hydrogenation,^128^ indicating an associative CO_2_ methanation process.^36^ However, as described earlier, higher temperatures (≥500 °C) thermodynamically favour the formation of CO rather than CH_4_. Additionally, η-Al_2_O_3_ alone can also form hydrogen carbonates species as starting intermediates (Fig. 3e), but Ni as the active phase provides hydrogen atoms necessary for each hydrogenation step.

### Ensuring sustainability through recycling spent NCts

Deactivation of NCts due to coke (carbon) formation or structural collapse from high-temperature sintering is often inevitable. Although no deactivation was observed in the 4 and 8 Ni/η-Al_2_O_3_ NCts during the study, it is important to consider reactivation for sustained CO_2_ methanation performance. Reactivation methods, such as coke removal through heating (800 °C) or chemical treatment, often lead to complete or partial structural collapse of the NCts. Thus, recycling spent Ni/η-Al_2_O_3_ NCts into Ni and Al precursors presents a more viable and sustainable option.

**Fig. 4:**
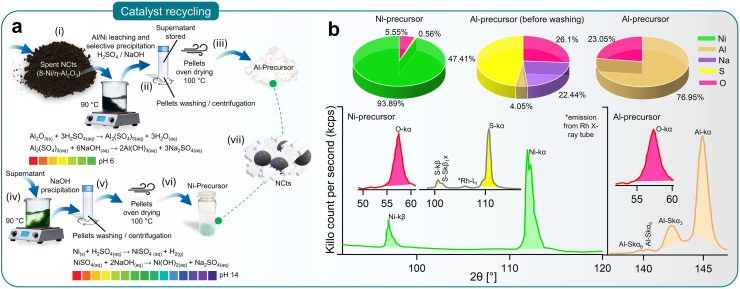
Recycling of spent NCts. (a) Schematics (steps i–vii) elaborating the recycling method of Al and Ni from spent Ni/η-Al_2_O_3_ NCts through acid leaching and selective NaOH precipitation (pH 0–14). (b) XRF spectroscopy showing elemental concentration (%) in Al and Ni precursors recovered from spent Ni/η-Al_2_O_3_ NCts (top) and characteristic XRF peaks related to Ni, Al, S and O (bottom).

Fig. 4a illustrates the recovery process of Al and Ni through acid leaching and selective NaOH precipitation (pH 0–14).^129^ The spent Ni/η-Al_2_O_3_ NCts, appearing as a black powder, were treated with H_2_SO_4_ to initiate the leaching of Ni and Al ions. This process was accelerated by heating the mixture to 90 °C for 2 hours, resulting in the formation of Al_2_(SO_4_)_3_ and NiSO_4_. Al_2_(SO_4_)_3_ was then recovered as Al(OH)_3_ by increasing the pH to 6 through NaOH treatment and centrifugation. The supernatant was stored for later Ni recovery. As shown in Fig. 4b, XRF spectroscopy confirmed that washing the recovered pellets three times with diH_2_O was necessary to remove Na_2_SO_4_ and fully recover Al as Al_2_O_3_, with predictable Al peaks (K_α_ and SK_α3,5,6_) visible in the XRF spectrum.

Further treatment of the supernatant at 90 °C with NaOH until pH 14 resulted in the precipitation of Ni(OH)_2_. The Ni(OH)_2_ pellets were washed and oven-dried to achieve a Ni/O purity of nearly 99%, with characteristics XRF peaks for Ni (K_α_, and K_β_) and O (K_α_), as shown in Fig. 4b. Although XRF spectroscopy is not considered reliable for lighter elements such as O, it effectively traced the Ni and Al concentrations^130^ (Fig. 4b). This method ensured the effective recovery of NiSO_4_, demonstrating the feasibility of recycling spent NCts into valuable precursors.

## Conclusions

In summary, our study demonstrates the successful upcycling of hazardous waste materials, specifically spent Ni-MH batteries and aluminium foil, into high-performance NCts for CO_2_ methanation. Nickel sulfate was extracted from battery waste and converted into Ni(OH)_2_ hydrogel complex, while waste aluminium foil was processed into alumina (Al_2_O_3_). The combination of Ni(OH)_2_ hydrogel complex with alumina resulted in the synthesis of Ni/η-Al_2_O_3_ NCts with 4 wt% and 8 wt% Ni loading. Thorough characterization by XRD, STEM, EFTEM, HRTEM, SAED, and EELS confirmed a disordered cubic structure of η-Al_2_O_3_ and its stability during CO_2_ hydrogenation. The 8% Ni variant demonstrated excellent catalytic performance, achieving 99.8% selectivity, 59% yield of CH_4_ at 400 °C and GHSV of 2984.16 h^−1^, although higher temperatures (>450 °C) led to increased CO production due to the RWGS reaction.

Further investigation using *operando* DRIFTS provided insights into the possible CO_2_ methanation mechanism over Ni/η-Al_2_O_3_ NCts. DRIFTS coupled with GC + MS revealed formation of key intermediates, such as hydrogen carbonates, formates, and methoxy species, illustrating the dynamic conversion of CO_2_ to CH_4_. Methane formation was observed above 300 °C, with higher Ni loading (8 wt%) enhancing CH_4_ production due to a combination of factors, including a larger number of Ni active sites per gram of catalyst and the influence of smaller (18–39 nm) Ni particle size. HRTEM analysis revealed that Ni nanoparticles in the 18–39 nm range were well-dispersed on the η-Al_2_O_3_ surface, ensuring a higher proportion of active surface atoms. This, possibly coupled with improved interaction between Ni and reactants, contributed to the enhanced CH_4_ yields at higher loadings. Moreover, the study also proposes an associative CO_2_ methanation pathway involving sequential adsorption and hydrogenation of CO_2_, with formate and methoxy intermediates leading to methane. At lower Ni loadings (4 wt%) or higher temperatures (450–550 °C), CO formation due to RWGS becomes more prevalent. To further validate the proposed mechanistic pathway, future studies could employ SSITKA-DRIFTS-MS, enabling isotopic labelling experiments to correlate the dynamics of surface and gas-phase species. Alternatively, MES could be applied. These would provide definitive evidence for the nature and role of reaction intermediates. Overall, these findings not only highlight the potential of waste-derived NCts for efficient CO_2_ methanation but also provide valuable insights into the reaction mechanisms involved, particularly the role of Ni over disordered η-Al_2_O_3_.

Finally, the successful recovery of Ni and Al precursors from Ni/η-Al_2_O_3_ NCts through acid leaching and selective NaOH precipitation highlights the sustainability and economic viability of this approach.

## Methods

### Synthesis procedures

The recovery of Ni from Ni-MH batteries, aluminium from aluminium foil, and the synthesis of NCts was accomplished through the following 12 steps, as illustrated in Fig. 1a and detailed below.

i. *Disassembly and washing of Ni-MH batteries*: the cylindrical spent Ni-MH batteries were disassembled to extract the cathode and anode components. The disassembled materials were then thoroughly washed with diH_2_O to remove the KOH electrolyte, continuing until the pH of the supernatant reached neutral (pH 7).

ii. *Leaching of nickel ions*: the cathode material (Ni(OH)_2_/NiOOH), appearing as a black coiled mat, was placed in a reaction beaker. Dilute H_2_SO_4_ was added to initiate the leaching of nickel ions. This process was accelerated by heating the mixture to 80 °C for 15 minutes. The reaction is represented by the following equation (eqn (1)):1
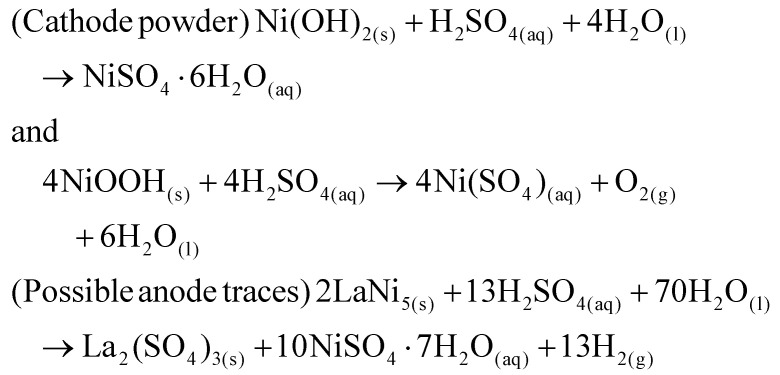


iii. *Separation and crystallization of NiSO*_*4*_: after cooling the suspension to room temperature, the precipitated La_2_(SO_4_)_3_ traces settled at the bottom, while the blue-green NiSO_4_·*x*H_2_O solution remained at the top. The NiSO_4_·*x*H_2_O solution was filtered using Whatman filter paper, heated to 100 °C to concentrate the mixture, and then cooled to collect crystallized NiSO_4_·7H_2_O.

iv. *Preparation of NiSO*_*4*_*solution and reduction to Ni(OH)*_*2*_: a 0.5 M solution of NiSO_4_·7H_2_O was prepared in diH_2_O, heated to 85 °C at 300 rpm, and 0.25 M l-glutamic acid was added. The mixture was then reduced to Ni(OH)_2_ using a 5 M NaOH solution, continuing until the pH reached 10.

v. *Washing and storage of Ni(OH)*_*2*_: the Ni(OH)_2_, forming a hydrogel, was washed with diH_2_O through centrifugation to remove uncoordinated reducing agents (l-glutamic acid/NaOH) and stored for further use.

vi. *Dissolution of waste Al-foil*: waste aluminium foil was dissolved according to the following equation (eqn (2)):

2 2Al_(s)_ + 6HCl_(aq)_ → 2AlCl_3(aq)_ + 3H_2(g)_

vii. *Reduction to Al(OH)*_*3*_*pellets*: the obtained AlCl_3_ was slowly reduced with 5 M NaOH to form Al(OH)_3_ pellets. This reaction was performed at 85 °C.

viii. *Recovery of Al(OH)*_*3*_*pellets*: after cooling the reaction mixture, the suspension was filtered using Whatman filter paper to recover the precipitated Al(OH)_3_ pellets.

ix. *Drying of Al(OH)*_*3*_*pellets*: the Al(OH)_3_ pellets were washed with diH_2_O through centrifugation and dried in an oven for 24 hours to obtain Al_2_O_3_ powder.

x. *Synthesis of NCts*: Ni(OH)_2_ and Al_2_O_3_ powder were mixed with Ni at weight percentages of 4% and 8%. The mixture was first stirred and then sonicated for 30 minutes.

xi. *Drying of the suspension*: the resultant suspension was oven-dried at 100 °C overnight.

xii. *Calcination of the dried powder*: the oven-dried powder was calcined at 550 °C (4 hours) to obtain 4% or 8% (wt%) Ni over η-Al_2_O_3_, denoted as 4-Ni/η-Al_2_O_3_ and 8-Ni/η-Al_2_O_3_, respectively.

Most importantly, the NCts preparation uses minimal H_2_SO_4_ and HCl to recover nickel and aluminium from waste, employing a closed system and effluent treatment ensuring environmental safety, aligning with green chemistry principles.^131^

### Characterization

The chemical composition and purity of the extracted Ni from spent Ni-MH batteries and spent NCts were determined using X-ray fluorescence spectrometry (XRF) on a PANalytical AxiosmAX WD-XRF™ system, equipped with a rhodium tube as the radiation source. XRF measurements were performed on pressed pellets containing approximately 10 wt% wax. The samples were irradiated with X-rays, causing the elements within the sample to emit secondary fluorescent X-rays. These emitted X-rays were detected and analyzed to identify the specific elements present and their relative abundances. Quantitative analysis was conducted using the fundamental parameter (FP) method, which corrects for matrix effects and provides accurate concentration values for each element. The data was processed to determine the weight percentages of the elements, enabling a detailed comparison of the elemental composition of the recovered Ni.

X-ray diffraction (XRD) measurements were conducted to elucidate the atomic structure of various crystalline phases, including metals, and oxides. Diffractograms were obtained using a PANanalytical X'Pert Pro™ Bragg–Brentano™ powder diffractometer at the X-ray Center of TU Wien, with Cu K-α radiation (wavelength of 1.54 Å) as the source. Small amounts of each catalyst, including calcined catalysts and those subjected to three different reduction temperatures, were applied to a silicon wafer Si (111) layer fixed to a sample holder. The positions (2*θ* angles) of the measured reflexes were compared with diffractograms from the ICDD International Centre for Diffraction Data™ database to identify the crystalline phases.

The morphology and crystal structure of the catalysts were analyzed using a FEI TECNAI G2 F20™ microscope at the University Service Center for Transmission Electron Microscopy (USTEM) at TU Wien. This microscope, equipped with a field emission gun (X-FEG) operating at 200 kV, was used to examine NCts samples loaded onto a carbon-coated Cu grid and inserted into the TEM's inlet system with a single tilt holder. Various TEM images, including high-angle annular dark field (HAADF), high-resolution (HR) TEM, energy-filtered (EF) TEM, and scanning (S) TEM, were recorded for each NCts both before and after the reaction. Structural alterations during the reaction were identified through image comparison, with the high resolution of HRTEM images allowing precise measurement of lattice planes to identify different phases. Additionally, selected area electron diffraction (SAED) was recorded for Ni and η-Al_2_O_3_ crystal structure analysis. Electron energy loss spectroscopy (EELS) measurements were also conducted to investigate elemental distribution. Finally, micrographs were analyzed using Digital Micrograph software (Gatan™).

Temperature programmed reduction (TPR) was employed to investigate the reducibility of NCts. The H_2_ TPR analysis was performed in a continuous fixed-bed quartz tube reactor. Approximately 50 mg of NCts was loaded into the reactor tube, which was then placed in a heating furnace. Gas flows of argon and hydrogen (the reducing gas) were precisely controlled using calibrated mass flow controllers. A total flow of 100 mL min^−1^ with 10 vol% H_2_ in Ar was passed through the sample. During the experiment, the furnace was heated from room temperature to 500 °C at a rate of 10 °C min^−1^. The quartz tube reactor was connected to a quadrupole mass spectrometer (Balzers Prisma™), which recorded the mass signals of H_2_ (*m*/*z* = 2) and H_2_O (*m*/*z* = 18) over time as a function of temperature. These experiments were conducted for both synthesized NCts and the pure η-Al_2_O_3_ support.

Brunauer–Emmett–Teller (BET) analysis of the as-prepared NCts was conducted using a Micromeritics surface area and porosity analyzer. To determine the specific surface area (SSA), N_2_ adsorption at −196 °C was performed on an ASAP 2020 Micromeritics™ apparatus with a 0.5 g sample, preheated under vacuum (<0.013 mbar) at 150 °C for 3 hours. The SSA was evaluated based on the linear portion of the BET analysis. Pore size distributions were obtained by applying the Barrett–Joyner–Halenda (BJH) equation to the desorption branch of the isotherm, and the total pore volume was estimated from the N_2_ uptake at a *P*/*P*^0^ of 0.99.

### CO_2_ methanation

For the kinetic measurements of the nanocatalysts (NCts), a pre-treatment process was conducted to ensure the removal of surface contaminants and activation of the NCts. Specifically, 20 mg of NCts was placed between quartz plugs inside the capillary tube, which was set up in the reactor and subjected to a 5% H_2_ in Ar atmosphere at 550 °C for 30 minutes, with a heating rate of 10 °C per minute (see the reactor setup in Fig. 2a). Following this pre-treatment, the temperature of the NCts bed was reduced to 250 °C, controlled precisely by a thermocouple. This step was crucial to prepare the catalyst for subsequent catalytic reactions by ensuring optimal surface conditions.

For the catalytic reaction, a gas mixture comprising 5% CO_2_, 20% H_2_, and 75% Ar was introduced at 1 bar pressure, with a total flow rate of 50 ml min^−1^. The catalytic activity was tested across a temperature range of 250 °C to 550 °C. Effluent gases were continuously analyzed using a gas chromatography/mass spectrometry (GC + MS) system, equipped with a capillary column designed for separating light hydrocarbons and permanent gases. The quadrupole mass spectrometer (QMS, Prisma Plus QMG 220, Pfeiffer Vacuum) operated in electron ionization (EI) mode to detect and quantify reaction products online, including methane (CH_4_) and carbon monoxide (CO). Additionally, a gas chromatograph (GC) from Agilent Technologies, equipped with a thermal conductivity detector (TCD) and a flame ionization detector (FID), was used for product analysis. Data acquisition was performed at regular intervals using Agilent Chemstation software (B.04.03), enabling real-time monitoring of catalytic performance under steady-state conditions. Retention times and mass spectral data were utilized to accurately identify the compounds formed during the reaction.

Before the experiments, the TCD and FID detectors was calibrated using standard gas mixtures to ensure precise quantification of the detected species. Calibration curves were generated by plotting the peak areas against the known concentrations of the standards (CO_2_, CO, CH_4_, and H_2_). Linear regression was employed to establish the relationship between peak area and concentration. During the experiments, the peak areas corresponding to various reactants and products were recorded. These peak areas were then used to determine the concentrations of the molecules present. The calibrated peak areas from GC chromatograms were utilized for calculation of the, *e.g.*, Conversion_CO_2__ (%) in eqn (3),3
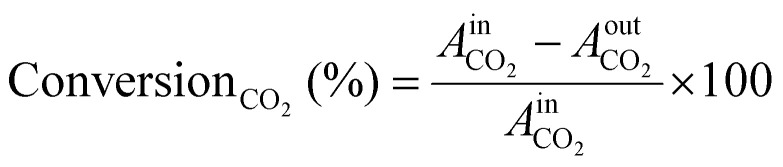
with, 
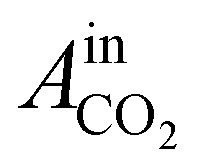
 = peak area of CO_2_ entering the reactor; 
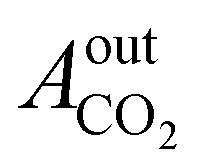
 = peak area of CO_2_ exiting the reactor.

The selectivity of the catalysts for producing CH_4_ Selectivity_CH_4__ (%) was determined using eqn (4),4
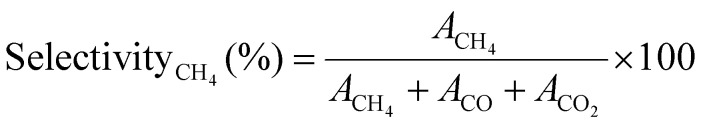
with, *A*_CH_4__ = peak area of CH_4_; *A*_CO_ = peak area of CO; *A*_CO_2__ = peak area of CO_2_.

Similarly, the CO Selectivity_CO_ (%) was determined using eqn (5)5
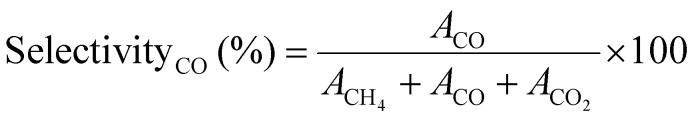
Using conversion (eqn (3)) and selectivity (eqn (4) or (5)), the yield of CH_4_ or CO can be calculated *via*eqn (6) and (7),6
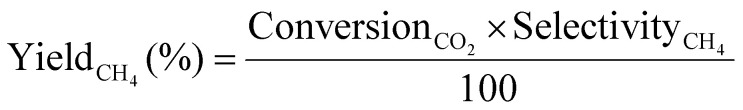
7
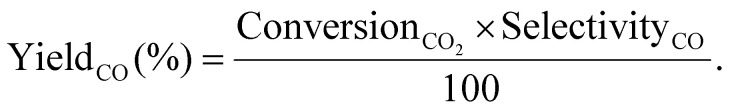


Furthermore, the GHSV can be calculated using eqn (8),8
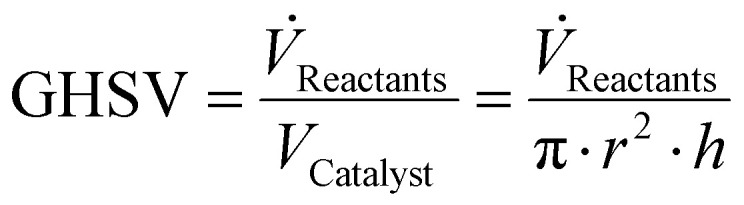
GHSV = Gas Hourly Space Velocity (h^−1^); *V̇*_Reactants_ = volumetric flow rate of reactants; *V*_Catalyst_ = volume of catalytic bed; *V*_Catalyst_ = volume of catalytic bed; *r* = internal radius of reactor; *h* = height of catalytic bed.

Moreover, the reciprocal value of the GHSV is the residence time (*τ*) (eqn (9)), so that9
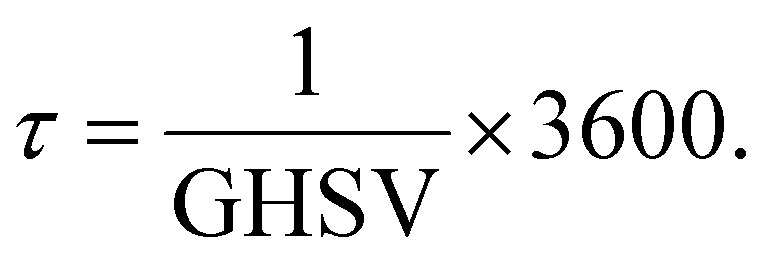


Finally, to determine the STY, first the volume of the reagent gas is calculated according to (eqn (10))

10 *V*_CO_2__ = *V̇*_CO_2__ × *τ*

*V̇*_CO_2__ = total gas flow of CO_2_; *τ* = residence time; *V*_CO_2__ = volume of CO_2_.

Then, using the residence time (*τ*) and the gas flow rate, the molar amount of the product, assuming that it equals that of the reagent gas, is determined by rearranging the universal gas equation (eqn (11)),11
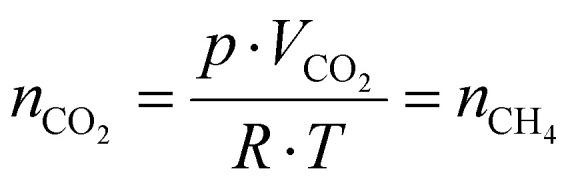
*p* = reaction pressure; *R* = universal gas constant; *T* = optimum reaction temperature; *n*_CO_2__ = amount of CO_2_; *n*_CH_4__ = maximum amount of produced CH_4_.

The STY values are finally obtained by inserting the measured CH_4_ in (eqn (12)),12
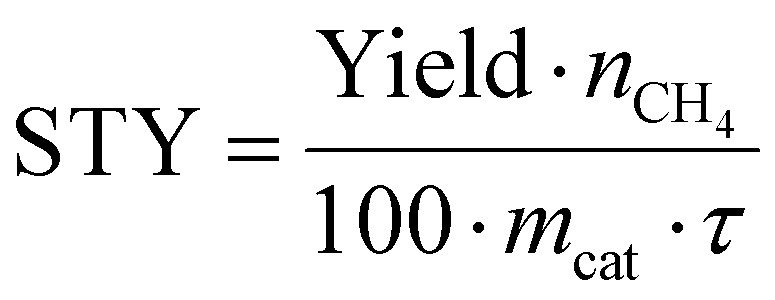
Yield = measured CH_4_ yield; *n*_CH_4__ = maximum amount of produced CH_4_; *m*_cat_ = mass of catalyst; *τ* = residence time.

Detailed calculations on GHSV, residence time (*τ*) and STY for 4 and 8-Ni/η-Al_2_O_3_ NCts can be found in Note 2 of the ESI.†

At last, the STY_CH_4__ of the best-performing NCts (*e.g.*, 8-Ni/η-Al_2_O_3_ at 400 °C) was compared with the STY_CH_4__ values of other Ni-based catalysts reported in the literature. To account for the varying Ni loading and reactions conditions, the literature STY_CH_4__ values were normalized (eqn (13)) to the same Ni content (%), pressure (1.01325 bar), and temperature (400 °C) for consistency.13
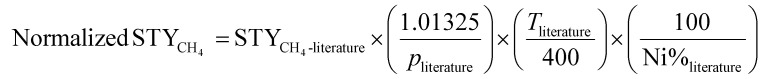
Normalized STY_CH_4__ = normalized space time yield of CH_4_ at 1.01325 bar per 400 °C (mmol_CH_4__ g_Ni_^−1^ h^−1^); STY_CH_4_-literature_ = space time yield of CH_4_ from literature (mmol_CH_4__ g_cat_^−1^ h^−1^); *p*_literature_ = pressure of reactor from literature (bar); *T*_literature_ = temperature of reactor from literature (°C); Ni%_literature_ = nickel content of catalyst from literature (%).

### *Operando* DRIFTS/GC + MS measurements

The CO_2_ methanation mechanism was explored using *operando* Diffuse Reflectance Infrared Fourier Transform Spectroscopy (DRIFTS), conducted with a Bruker Vertex 70 spectrometer. The reaction chamber, equipped with CaF_2_ windows, enabled the passage of infrared light through the sample while maintaining controlled gas flow and temperature conditions. Initially, the NCts samples were loaded into the chamber and subjected to pre-treatment under a 5% H_2_/Ar flow at 550 °C for 30 minutes, with a heating rate of 10 °C min^−1^. Following this pre-treatment, the samples were cooled to the desired reaction temperature, and the gas flow was switched to a CO_2_ and H_2_ mixture (1 : 4 ratio) at a total flow rate of 50 mL min^−1^, simulating CO_2_ hydrogenation conditions. Additionally, for a temperature-programmed desorption study of the surface adsorbed molecules, the sample (η-Al_2_O_3_) was heated in Ar to 480 °C (10 °C min^−1^) in the DRIFTS cell while recording IR spectra. During the DRIFTS experiments, spectra were collected using OPUS 6.5 software at a resolution of 2 cm^−1^, with 128 scans recorded per spectrum. A background spectrum was recorded under pure Ar flow at the reaction temperature, and all spectra were normalized against this background to isolate signals from adsorbed species on the catalyst surface. The spectral region of interest (4000–1000 cm^−1^) was analyzed to monitor the formation and evolution of surface intermediates throughout the reaction. Additionally, the reactor effluent was continuously analyzed by GC + MS to ensure that the DRIFTS observations were consistent with catalytic performance. This approach allowed for direct correlation of the *operando* DRIFTS data with catalytic activity and selectivity under real reaction conditions.

### Recycling of spent NCts

In steps i–iii (Fig. 4a), the spent Ni/η-Al_2_O_3_, appearing as a black powder, were placed in a reaction beaker where H_2_SO_4_ was added to initiate the leaching of Ni ions. This process was accelerated by heating the mixture to 90 °C for 2 hours. The reactions are represented by the following equations:

14 Al_2_O_3(s)_ + 3H_2_SO_4(aq)_ → Al_2_(SO_4_)_3_(aq) + 3H_2_O_(aq)_

and

15 Ni_(s)_ + H_2_SO_4(aq)_ → NiSO_4(aq)_ + H_2(g)_.

Subsequently, Al_2_(SO_4_)_3_ was recovered as Al(OH)_3_ by increasing the pH to 6 through NaOH treatment, followed by washing with diH_2_O and centrifugation. The supernatant was stored for later Ni recovery, and the washed Al pellets were oven-dried at 100 °C to obtain the Al-precursor. This reaction is represented by the equation:

16 Al_2_(SO_4_)_3(aq)_ + 6NaOH_(aq)_ → 2Al(OH)_3(aq)_ + 3Na_2_SO_4(aq)_.

In steps iv–vii (Fig. 4a), the supernatant containing NiSO_4_ was further treated at 90 °C with NaOH until the pH reached 14, resulting in the precipitation of Ni(OH)_2_. This process is represented by the equation:

17 NiSO_4(aq)_ + 2NaOH_(aq)_ → Ni(OH)_2(aq)_ + Na_2_SO_4(aq)_.

The Ni precipitation was followed by washing with diH_2_O through centrifugation. The washed Ni pellets were then oven-dried at 100 °C to obtain the Ni-precursor. Both the Al and Ni precursors can subsequently be used to synthesize Ni/η-Al_2_O_3_ NCts.

## Author contributions

Qaisar Maqbool: conceptualization, methodology, validation, software, formal analysis, investigation, data curation, writing – original draft, writing – review & editing. Hamilton Uchenna Aharanwa: methodology, validation, software, formal analysis, investigation, data curation, writing – review & editing. Michael Stöger-Pollach: methodology, validation, formal analysis, investigation, writing – review & editing. Günther Rupprechter: conceptualization, validation, resources, writing – review & editing, supervision.

## Data availability

The data supporting this article have been included as part of the ESI.†

## Conflicts of interest

There are no conflicts of interest to declare.

## Supplementary Material

GC-027-D4GC05217J-s001
