# Supplementary material for: Upcycling hazardous waste into high-performance Ni/η-Al2O3 catalysts for CO2 methanation
Source: Green Chem. 2025 Feb 7;27(10):2706–22. doi: 10.1039/d4gc05217j (PMC11826383; doi:10.1039/d4gc05217j)
Supplement: GC-027-D4GC05217J-s001 [file GC-027-D4GC05217J-s001.pdf]

Supplementary Information (SI) for Green Chemistry.
This journal is © The Royal Society of Chemistry 2025

Electronic Supplementary Information (ESI) for

Upcycling Hazardous Waste into High-Performance Ni/η-Al_2O_3 Catalysts for CO_2 Methanation

Qaisar Maqbool[1], Hamilton Uchenna Aharanwa[1], Michael Stöger-Pollach[2], Günther Rupprechter[1]*

[1]Institute of Materials Chemistry, TU Wien, Getreidemarkt 9/BC, 1060 Vienna, Austria.
[2]University Service Center for Transmission Electron Microscopy, TU Wien, Stadionallee 2/057-02, 1020 Vienna, Austria.

*Correspondence: guenther.rupprechter@tuwien.ac.at

Supplementary note 1:

The surface area of an average spherical NiO particle (as shown in Fig. S5) with a 6.5 nm diameter was calculated based on the following equation

$$A = 4\pi r^2$$

r = sphere radius (3.25 nm)
A = sphere surface area (132.7 nm^2)

i. The volume of the average particle was obtained based on:

$$V = \frac{4}{3}\pi r^3$$

V= 143.8 nm^3 = 143.8e-21 cm^3 = 1.44e-19 cm^3
ρ = NiO density (6.67 g/cm^3)

ii. The maass of the average particle was calculated using:

$$m = \rho * V$$

m = mass of the average NiO particle (9.6e-19 g)

iii. In the next step, the weight of NiO per gram of the catalyst was obtained:

$$m_{NiO} = \frac{\%_{Ni} * M_{NiO}}{M_{Ni}}$$

m_{NiO} = NiO mass (g)
$\%_{Ni}$ = Ni percentage (%)
M_{NiO} = NiO molar mass (g/mol) (74.7 g/mol)
M_{Ni} = Ni molar mass (g/mol) (58.7 g/mol)

iv. Given the obtained total NiO weight, the number of NiO particles per gram of catalyst was calculated:

$$N_{NiO} = \frac{m_{NiO}}{m}$$

N_{NiO} = number of NiO nanoparticles per gram catalyst (1.06e+17 for 8% Ni or 5.31e+16 for 4% Ni)

v. Finally, the total surface area of the NiO nanoparticles per gram catalyst was obtained:

$$SS_{NiO} = N_{NiO} * A$$

SS_{NiO} = NiO surface area (nm^2) per gram catalyst (1.41e+17 nm^2 for 8% Ni or 7e+16 nm^2 for 4% Ni, 14.1 m^2 for 8% Ni or 7.0 m^2 for 4% Ni)

Supplementary note 2:

Gas Hourly Space Velocity (GHSV) and Residence Time (τ)

To measure the interaction between the reactants (CO_2/H_2) and the catalyst bed in the reactor, it is necessary to calculate the gas hourly space velocity (GHSV). The GHSV describes the volume of reactants that passes through the catalyst bed per hour. Therefore, the total volumetric flow of the reactants, as well as the volume of the catalyst bed, are required for its calculation, as shown in the equation below,

$$\mathrm{GHSV} = \frac{\dot{\mathrm{V}}_{\mathrm{Reactants}}}{\mathrm{V}_{\mathrm{Catalyst}}} = \frac{\dot{\mathrm{V}}_{\mathrm{Reactants}}}{\pi \cdot \mathrm{r}^2 \cdot \mathrm{h}}$$

GHSV = Gas Hourly Space Velocity (h^{-1})

$\dot{V}_{Reactants}$ = Volumetric flow rate of reactants (0.75 L/h)

$V_{Catalyst}$ = Volume of catalytic bed ($2.51 \cdot 10^{-4}$ L)

r = internal radius of reactor (0.04 dm)

h = height of catalytic bed (0.05 dm)

GHSV = 2984.16 h^{-1}

Moreover, the reciprocal value of the GHSV is the residence time (τ), so that

$$\tau = \frac{1}{GHSV} \cdot 3600$$

meaning that a reactant molecule spends 1.21 seconds over the catalyst bed before leaving the reactor.

Space-Time Yield (STY)

The space-time yield (STY) is another parameter used to characterize the performance of a catalyst. To determine this, the volume of the reagent gas is calculated according to equation **(S1)**, using the residence time and the gas flow rate. The molar amount of the product, assuming that it equals that of the reagent gas, is determined by rearranging the universal gas equation **(S2)**. The STY values are finally obtained by inserting the measured CH_4 yield into equation **(S3)**.

$$\mathrm{V}_{CO_2} = \dot{\mathrm{V}}_{CO_2} \cdot \tau \qquad \textbf{(S1)}$$

$\dot{V}_{CO_2}$= total gas flow of CO_2 (2.5 mL/min = 0.15 L/h)

τ = residence time (0.0003 h)

V_{CO_2} = volume of CO_2 (0.000050 L)

$$n_{CO_2} = \frac{p \cdot \mathrm{V}_{CO_2}}{\mathrm{R} \cdot T} = n_{CH_4} \qquad \textbf{(S2)}$$

p = reaction pressure (1 atm = 1.01325 bar)

R = universal gas constant (0.083143 $L \cdot bar \cdot mol^{-1} \cdot K^{-1}$)

T = optimum reaction temperature (673 K)

n_{CO_2} = amount of CO_2 (0.0000009 mol)

n_{CH_4} = maximum amount of produced CH_4 (0.0000009 mol = 0.0009102 mmol)

$$STY = \frac{Yield \cdot n_{CH_4}}{100 \cdot m_{cat} \cdot \tau} \qquad \textbf{(S3)}$$

Yield = measured CH_4 yield (4-Ni/η-Al_2O_3 - 16.09 %, 8-Ni/η-Al_2O_3 - 59.16 %)

n_{CH_4} = maximum amount of produced CH_4 (0.0009102 mmol)

m_{cat} = mass of catalyst (0.02 g)

τ = residence time (0.0003 h)

STY = space-time yield (4-Ni/η-Al_2O_3 - 21.8562 $mmol_{CH_4} \cdot g_{cat}^{-1} \cdot h^{-1}$, 8-Ni/η-$Al_2O_3$ - 80.3460 $mmol_{CH_4} \cdot g_{cat}^{-1} \cdot h^{-1}$)

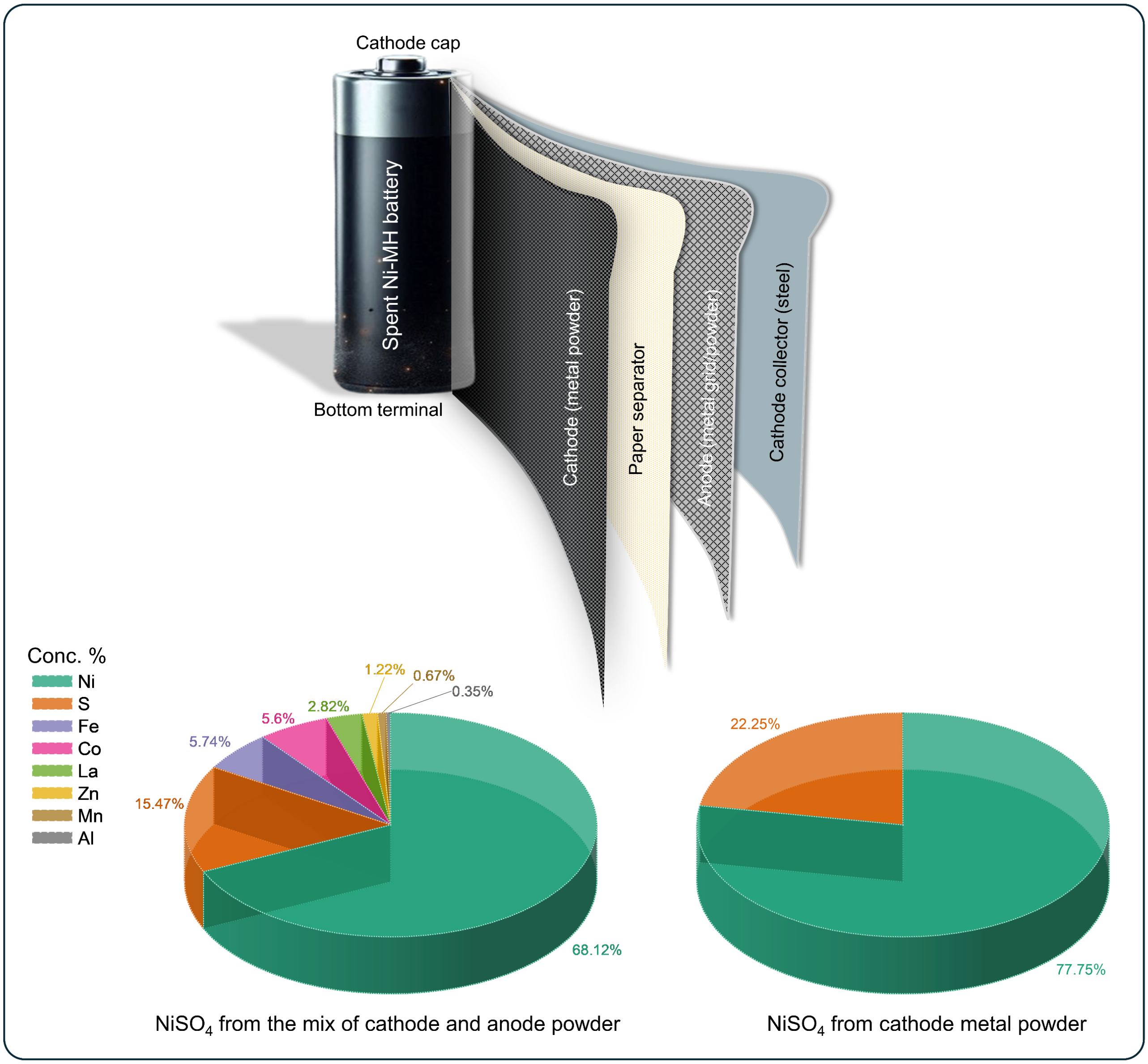

Supplementary Figure S1. Illustration showing dissected inner components of a Ni-MH battery (top). Concentration (%) of elements detected by XRF spectrometry of $NiSO_4$ (precursor) recovered from cathode and mix of cathode and anode parts of spent Ni-MH battery (bottom).

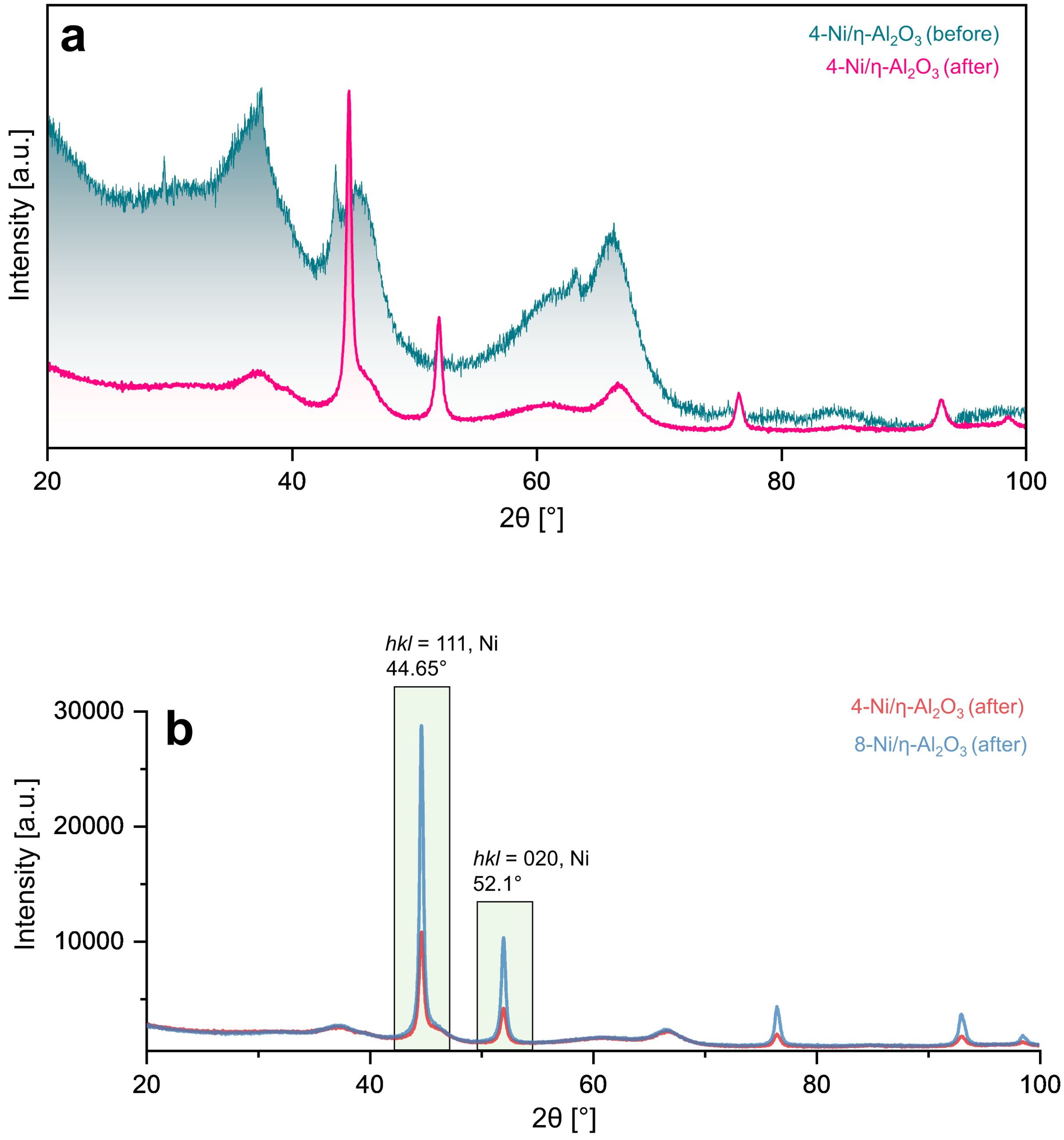

Supplementary Figure S2. (a) Crystal structure of 4-Ni/η-Al_2O_3 NCts before and after CO_2 methanation. (b) XRD comparison of 4 and 8-Ni/η-Al_2O_3 NCts after CO_2 methanation. The two most intense peaks were used to calculate the average Ni crystallite size (see main text for details).

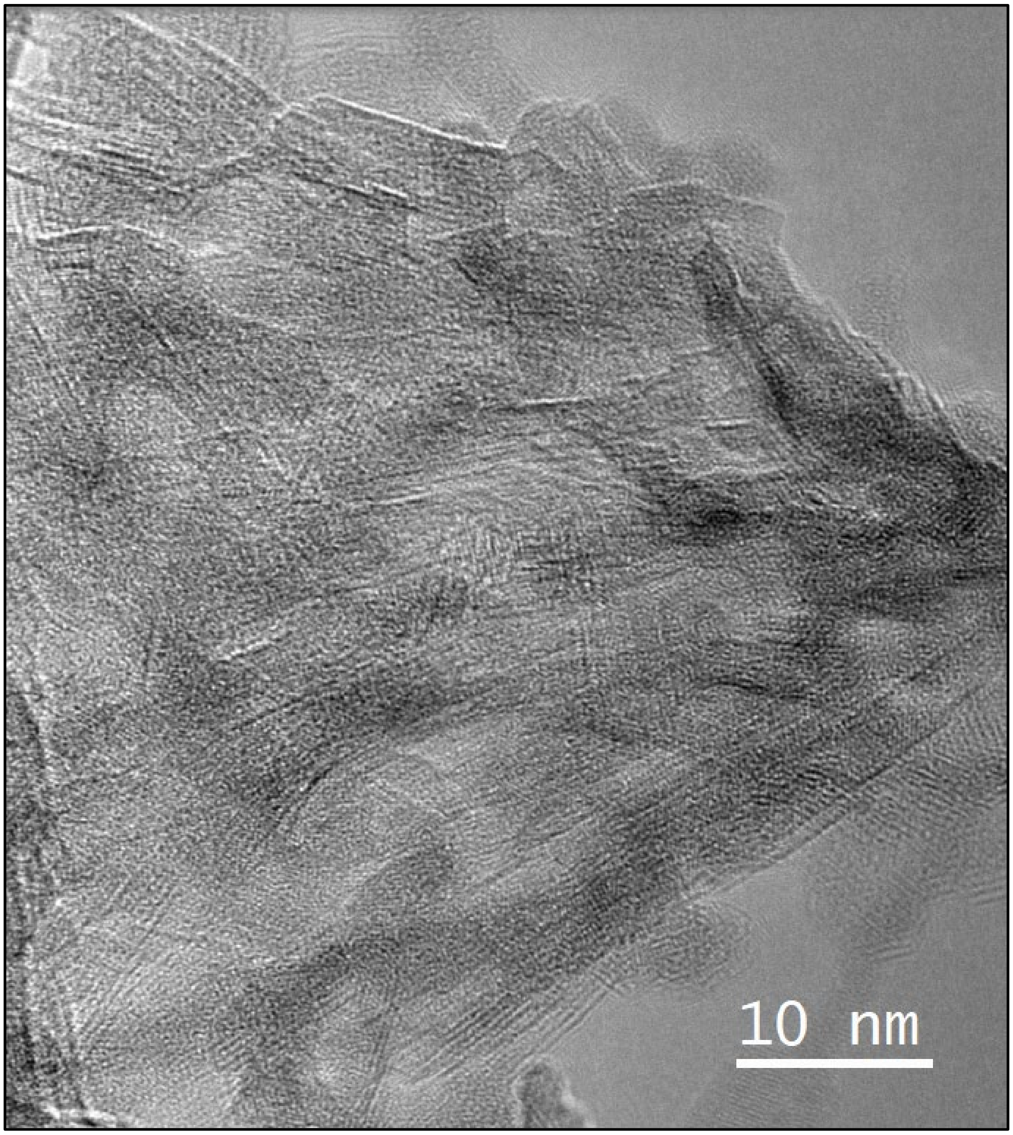

Supplementary Figure S3. HRTEM of η-Al_2O_3 support.

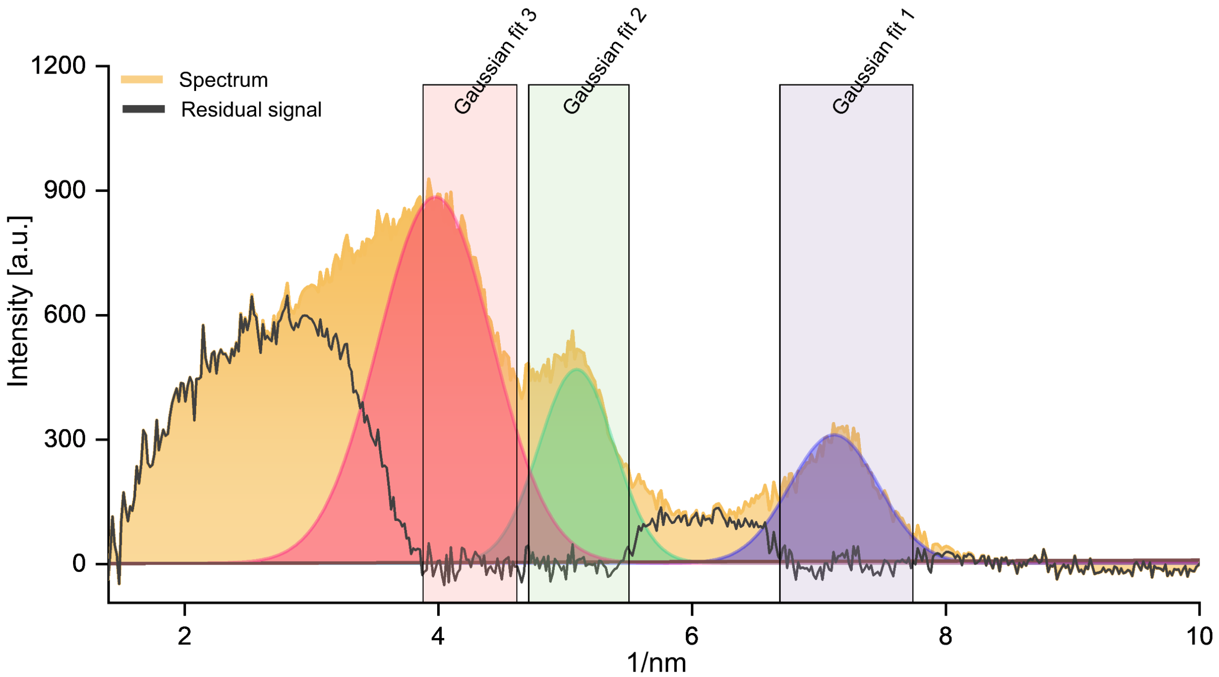

Supplementary Figure S4. Rotational average plot of the η-Al_2O_3 SAED (see inset of Fig. 1c, main text).

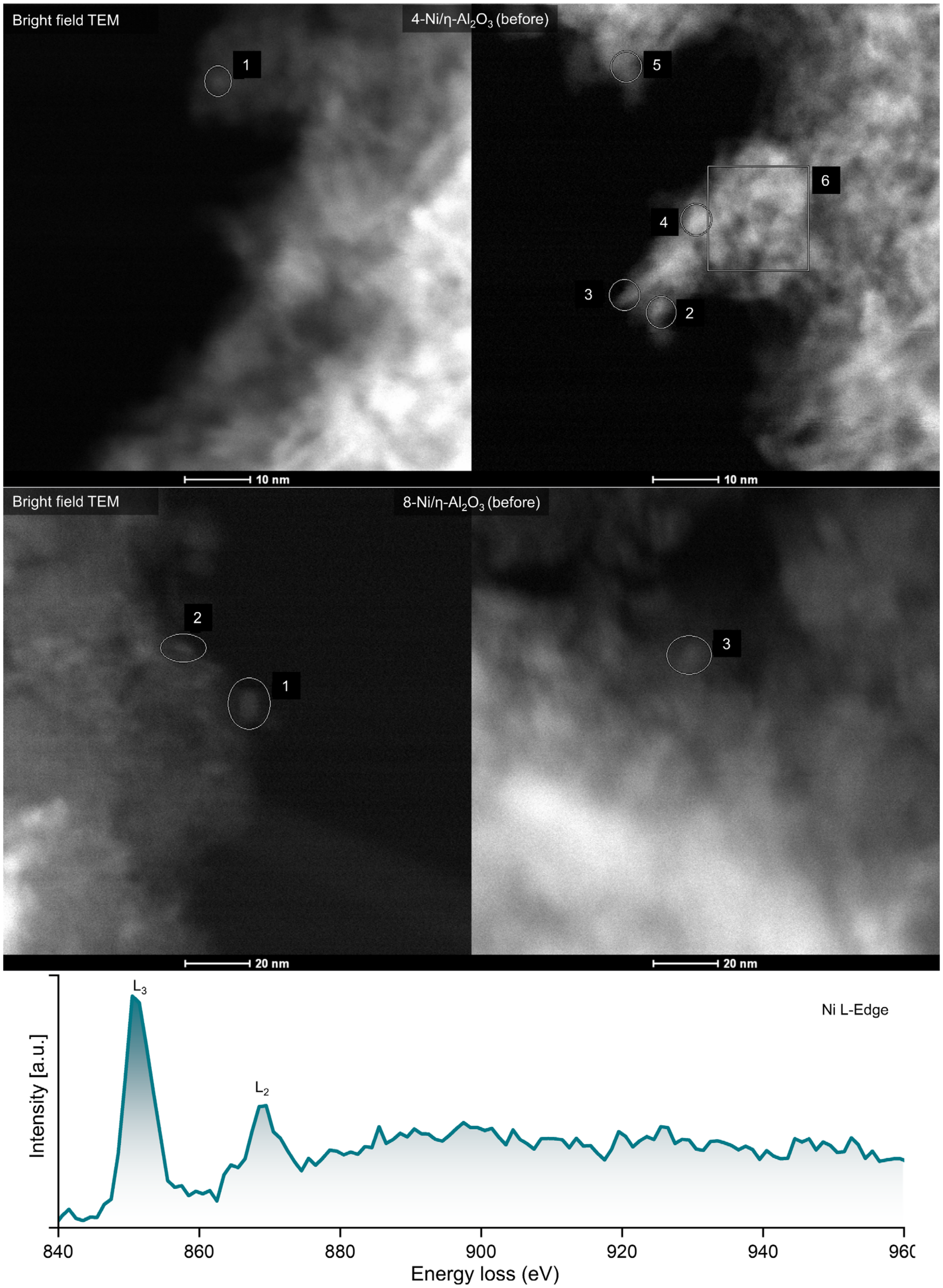

Supplementary Figure S5. Bright-field TEM images (top) of pristine 4-Ni/η-Al_2O_3 and 8-Ni/η-Al_2O_3 showing distribution of NiO within the η-Al_2O_3 matrix. The NiO in η-Al_2O_3 was identified based on the Ni L-edge using EELS (bottom).

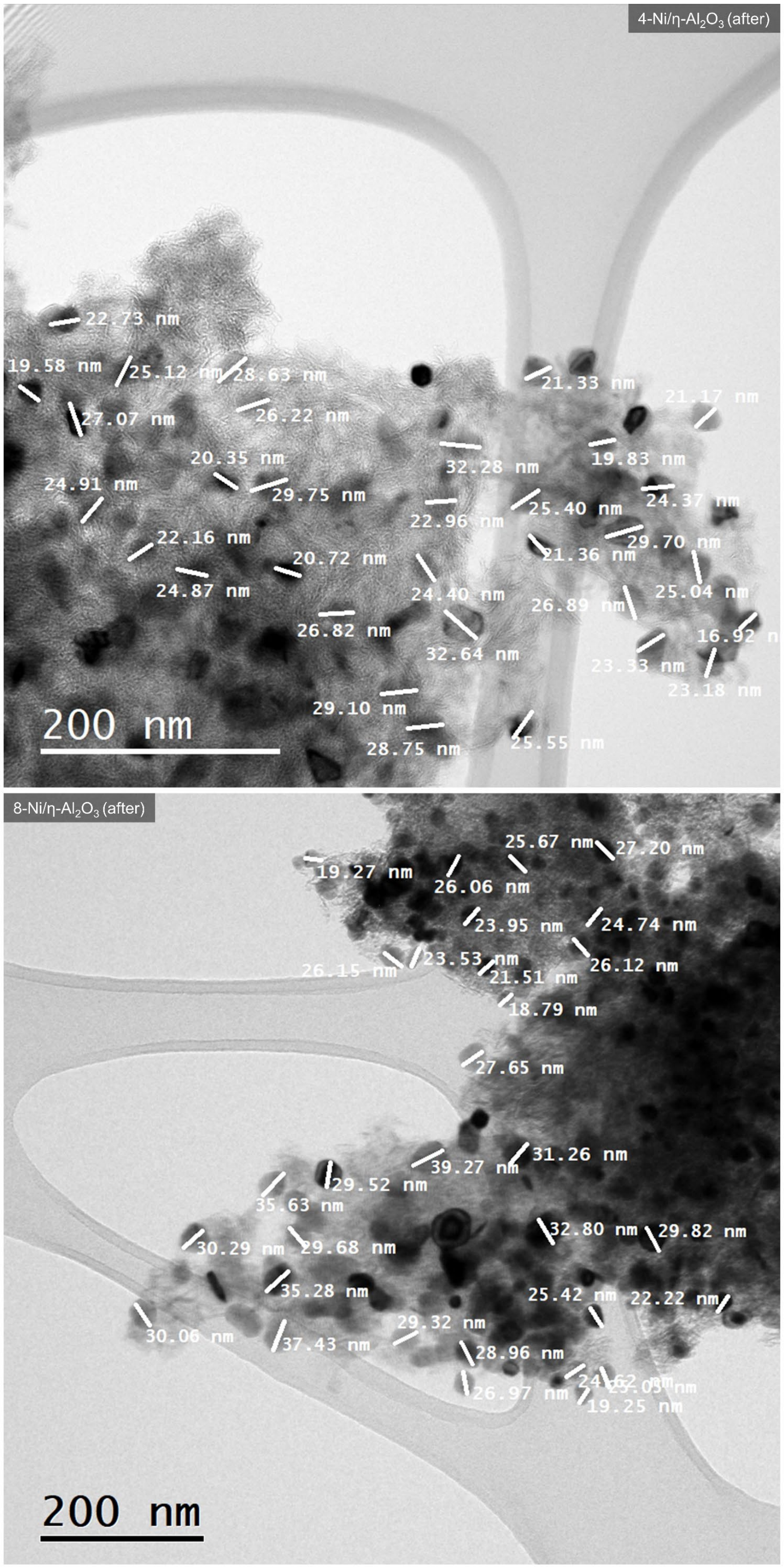

Supplementary Figure S6. TEM images demonstrating the homogenous Ni particle size and distribution in 4 and 8-Ni/η-Al_2O_3 after CO_2 methanation.

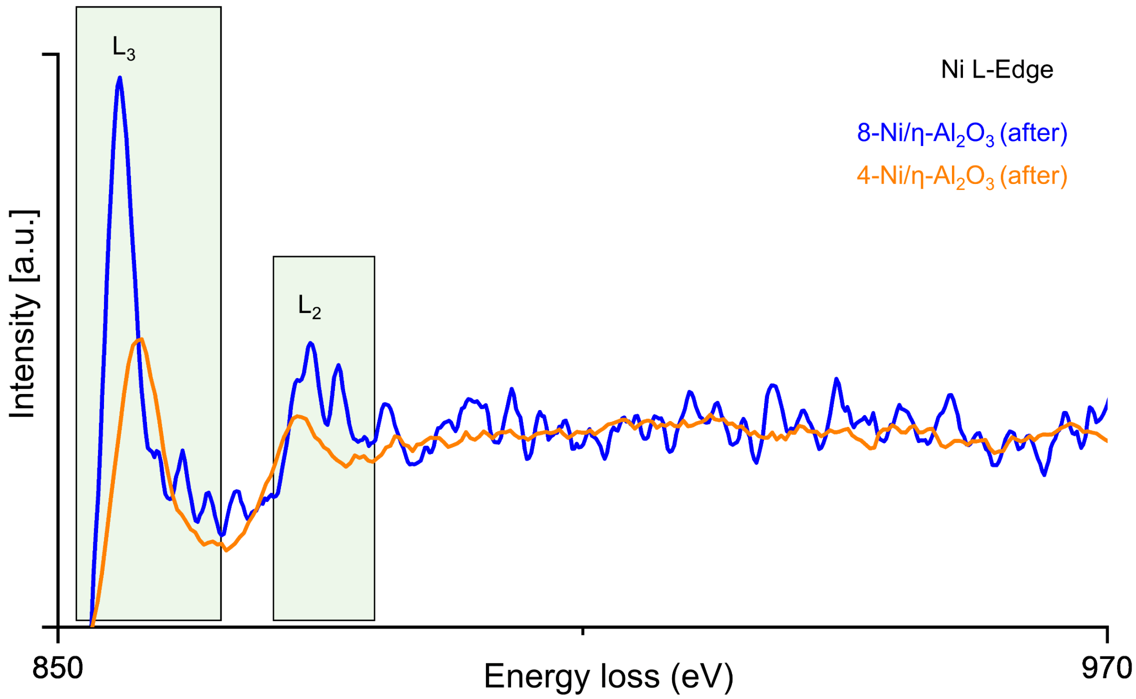

Supplementary Figure S7. EELS spectra corresponding to Ni $L_{3,2}$-edges of 4-Ni/η-Al_2O_3 and 8-Ni/η-Al_2O_3 after CO_2 methanation.

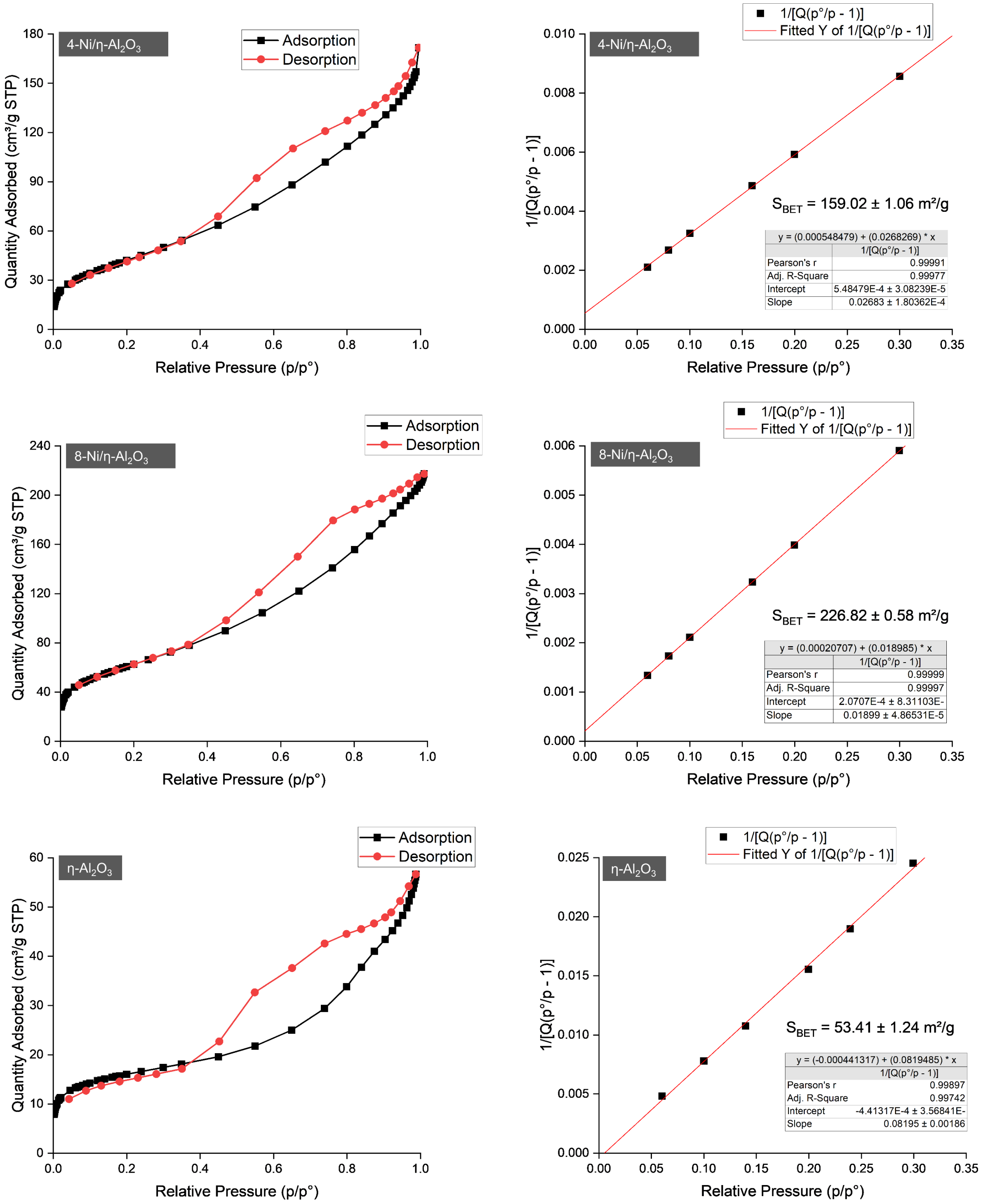

Supplementary Figure S8. BET surface area and BJH pore size analysis of 4, 8-Ni/η-Al_2O_3, and η-Al_2O_3.

Supplementary Table S1. Comparison of STY ($mmol_{CH_4}$. g_{Ni}^{-1}. h^{-1}) of best performing NCts (8-Ni/η-Al_2O_3) with Ni-based NCts from literature.

Materials description	Synthesis method	Catalytic conditions			Normalized STY $mmol_{CH_4}$. g_{Ni}^{-1}. h^{-1}	Supp. Refs.
		Pretreatment	Reaction (Gases%)	Pressure/ Temp.		
Ni-siliceous MCM-41	Hydrothermal Method	Reduced in pure H_2 at 700 °C for 0.5 h	H_2:CO_2 = 2.6:1	1.01325 bar /400°C	1315.46	1
Ni-CeO_2 nanorods	Hydrothermal Method	None	H_2:CO_2 = 4	1 bar /275°C	154.31	2
Ni-Microporous Graphene like Carbon	Incipient wetness impregnation method	Reduced in pure H_2 at 400 °C for 2 h	H_2:CO_2:N_2 = 4:1:1	1.01325 bar /400°C	858.69	3
Ni-Zn/SiO_2	Ammonia evaporation process	Reduced in pure H_2 at 500 °C for 3 h	H_2:CO_2 = 4	1.01325 bar /230°C	13.77	4
5-Ni/ZrO_2	Incipient wetness impregnation method	Reduced in pure H_2 at 500 °C for 5 h	H_2:CO_2:N_2 = 4:1:5	10 bar /300°C	189.51	5
Ni-zeolite from coal gangue	Wet impregnation	Reduced in pure H_2 at 500 °C for 1 h	15% CO_2, 60% H_2, and 25% Ar (molar basis)	1 bar /450°C	98.98	6
Ni/Mn-CeO_2	Sol-gel method	Reduced in pure H_2 at 550 °C for 2 h	H_2:CO_2 = 4	1.01325 bar /370°C	188.34	7
Ni-MgO/MgH_2	Mechanochemical ball-milling method	Reduced in H_2/N_2 gas (20% H_2) at 380 °C for 5 h	H_2:CO_2 = 4	10 bar /300°C	68.85	8
8-Ni/η-Al_2O_3	Battery waste-upcycling	Reduced in 5% H_2 at 550 °C for 30 min.	5% CO_2, 20% H_2, and 75% Ar	1 bar /400°C	1004.32	This work

Supplementary References

1 G. Du, S. Lim, Y. Yang, C. Wang, L. Pfefferle and G. L. Haller, *J. Catal.*, 2007, **249**, 370–379.
2 G. Varvoutis, M. Lykaki, S. Stefa, V. Binas, G. E. Marnellos and M. Konsolakis, *Appl. Catal. B Environ.*, 2021, **297**, 120401.
3 J. Wu, Z. Jin, B. Wang, Y. Han, Y. Xu, Z. Liang and Z. Wang, *Ind. Eng. Chem. Res.*, 2019, **58**, 20536–20542.
4 X. Chen, S. Ullah, R. Ye, C. Jin, H. Hu, F. Hu, Y. Peng, Z. H. Lu, G. Feng, L. Zhou and R. Zhang, *Energy and Fuels*, 2023, **37**, 3865–3874.
5 F. Lange, U. Armbruster and A. Martin, *Energy Technol.*, 2015, **3**, 55–62.
6 S. Bahraminia and M. Anbia, *Int. J. Hydrogen Energy*, 2024, **83**, 842–855.
7 Q. Fan, S. Li, L. Zhang, P. Wang and S. Wang, *J. Catal.*, 2022, **414**, 53–63.
8 H. Chen, P. Liu, J. Liu, X. Feng and S. Zhou, *J. Catal.*, 2021, **394**, 397–405.
